# In vitro synergy screens of FDA-approved drugs reveal novel zidovudine- and azithromycin-based combinations with last-line antibiotics against *Klebsiella pneumoniae*

**DOI:** 10.1038/s41598-023-39647-9

**Published:** 2023-09-02

**Authors:** Marta Gómara-Lomero, Ana Isabel López-Calleja, Antonio Rezusta, José Antonio Aínsa, Santiago Ramón-García

**Affiliations:** 1 Department of Microbiology. Faculty of Medicine, University of Zaragoza, C/ Domingo Miral S/N, 50009 Zaragoza, Spain; 2 Servicio de Microbiología, Hospital Universitario Miguel Servet, Zaragoza, Spain; 3 CIBER Respiratory Diseases, Carlos III Health Institute, Madrid, Spain; 4 Research and Development Agency of Aragon (ARAID) Foundation, Zaragoza, Spain

**Keywords:** Antimicrobial resistance, Drug screening, Clinical microbiology

## Abstract

Treatment of infections caused by multi-drug resistant (MDR) enterobacteria remains challenging due to the limited therapeutic options available. Drug repurposing could accelerate the development of new urgently needed successful interventions. This work aimed to identify and characterise novel drug combinations against *Klebsiella pneumoniae* based on the concepts of synergy and drug repurposing*.* We first performed a semi-qualitative high-throughput synergy screen (sHTSS) with tigecycline, colistin and fosfomycin (last-line antibiotics against MDR Enterobacteriaceae) against a FDA-library containing 1430 clinically approved drugs; a total of 109 compounds potentiated any of the last-line antibiotics. Selected hits were further validated by secondary checkerboard (CBA) and time-kill (TKA) assays, obtaining 15.09% and 65.85% confirmation rates, respectively. Accordingly, TKA were used for synergy classification based on determination of bactericidal activities at 8, 24 and 48 h, selecting 27 combinations against *K. pneumoniae*. Among them, zidovudine or azithromycin combinations with last-line antibiotics were further evaluated by TKA against a panel of 12 MDR/XDR *K. pneumoniae* strains, and their activities confronted with those clinical combinations currently used for MDR enterobacteria treatment; these combinations showed better bactericidal activities than usual treatments without added cytotoxicity. Our studies show that sHTSS paired to TKA are powerful tools for the identification and characterisation of novel synergistic drug combinations against *K. pneumoniae*. Further pre-clinical studies might support the translational potential of zidovudine- and azithromycin-based combinations for the treatment of these infections.

## Introduction

Antimicrobial resistance (AMR) is a major global public health problem. There were an estimated 4.95 million deaths worldwide associated with bacterial AMR in 2019, including 1.27 million deaths directly attributable to bacterial AMR^1^. In the European Union (EU), AMR is responsible for about 33,000 deaths and 1.1 billion Euros of costs to EU health care systems each year^2^. Carbapenemase-producing *Klebsiella pneumoniae* causes severe infections associated with 23–75% mortality rates^3^. In fact, it is considered among the most concerning pathogens with worldwide prevalence of carbapenem resistance infections increasing more than 7-fold in the EU since 2006. In addition, resistance to two or more antimicrobial groups is a growing concern^4, 5^.

The emergence of multidrug-resistant (MDR) and extensively drug-resistant (XDR) enterobacteria to most available antibiotics severely limits therapeutic options of infections caused by these pathogens. Clinicians are thus forced to use last-line antibiotics in combination therapy with uncertain success rates^6, 7^. In 2017, WHO highlighted drug-resistant enterobacteria as priority pathogens for which new antibiotics were urgently required^8^; however, the traditional process of drug discovery and development is long and costly^9, 10^. Although a few new clinical options have been recently approved, scarce evidence and lack of randomized clinical trials prevent from establishing optimal clinical guidelines. New strategies changing the current paradigm are urgently needed^11^.

Drug repurposing is an attractive approach that allows faster clinical implementation, since pharmacokinetic and pharmacodynamic (PKPD) parameters and toxicity packages are already defined in commercialized drugs^12^. The search for synergistic interactions has also emerged as a favourable approach to enhance the activity of drugs in combinatorial therapy in two different manners: first, expanding the therapeutic range of drugs whose potential use may be limited by toxicity issues and, second, by rescuing antimicrobials that do not reach efficacy breakpoints. Thus, synergistic combinations might show similar efficacy at lower, non-toxic doses. In addition, the use of combinatorial therapy might prevent the development of resistance derived from monotherapy^13^.

In this study, we aimed to identify partners that could potentiate the activity of tigecycline, colistin and fosfomycin, three last-line antibiotics currently used to treat infections caused by MDR *K. pneumoniae.* For this, we performed a semi high-throughput synergy screen (sHTSS) of a library of U.S. Food and Drug Administration (FDA) approved compounds in combination with tigecycline, colistin and fosfomycin^14^. Lead combinations were validated by checkerboard (CBA) and time-kill (TKA) assays. Synergistic and killing effects were evaluated at different time points to obtain a priority list of combinations. Among them, zidovudine and azithromycin resulted in the most potent synergistic partners of the three antimicrobials with the highest potential for clinical translation. Combinations based on zidovudine and azithromycin were further characterised for cytotoxicity and in vitro activity against a panel of antibiotic-resistant *K. pneumoniae* strains; their activities were also compared with usual last-resort combination treatments for MDR enterobacteria and proved to be superior.

## Materials and methods

### Bacterial strains, media and chemicals

The *K. pneumoniae* ATCC 13883 strain was used for the sHTSS and first validation step by CBA. For the characterisation of zidovudine- or azithromycin-based combinations, a well-characterised set of 12 *K. pneumoniae* strains (eight from clinical isolates and four from quality assessment exercises), classified as MDR/XDR^15^, was provided by the *Miguel Servet* University Hospital (Zaragoza, Spain) (Table S1). Bacterial identification was performed by matrix-assisted laser desorption/ionization time-of-flight (MALDI-TOF) mass spectrometry (Bruker Daltonik GmbH, Germany) and antimicrobial susceptibility by an automated broth microdilution method (Microscan Walkaway^®^, Beckman Coulter, Spain). Phenotypic detection of Extended-Spectrum β-lactamase (ESBL), AmpC, carbapenemases and colistin resistance was done according to European Committee on Antimicrobial Susceptibility Testing (EUCAST) guidelines^16^. Genotypic characterisation of the resistance mechanisms was performed at the National Microbiology Centre (Majadahonda, Spain).

Bacterial stocks (15% glycerol) were preserved at – 20 °C in Luria–Bertani (LB) broth. A new stock was thawed for every experiment to ensure assay robustness, and sub-cultured on Mueller Hinton broth (MHB) for 24 h before performing each Minimum Inhibitory Concentration (MIC), CBA and TKA assay. Drug susceptibility testing in solid media and sHTSS were performed in Mueller–Hinton agar (MHA). All cultures were incubated at 36–37 °C.

The FDA-approved drug library was purchased from Selleckchem (catalogue #L1300). Drugs were purchased from Sigma-Aldrich (Darmstadt, Germany), except tigecycline and ceftazidime (European Pharmacopoeia, Strasbourg, France), meropenem (Fresenius Kabi), ertapenem (MSD) and avibactam (AdooQ BioScience, Irvine, USA). Stock solutions were prepared fresh on the same day of plate inoculation after drug solubilization in DMSO or water.

### Drug susceptibility testing

1. Determination of MIC on liquid media was performed by broth microdilution in cation-adjusted MHB (CAMHB) according to Clinical & Laboratory Standards Institute (CLSI) guidelines^17^ by using the MTT [3-(4,5-dimethylthiazol-2-yl)-2,5-diphenyl tetrazolium bromide] assay^14, 18^. Briefly, two-fold serial dilutions of drugs were inoculated with 5 × 10^5^ Colony Forming Units (CFU) per mL (OD_550_ = 0.2 corresponded to 1.5 × 10^8^ cell/mL) in 96-well plates (V_F_ = 150 µL/well) and incubated for 18–20 h. For fosfomycin and ceftazidime-avibactam susceptibility tests, CAMHB was supplemented with 25 mg/L of glucose-6-phosphate and 4 mg/L of avibactam, respectively, according to EUCAST guidelines^19^. After incubation, 30 μL/well of 5 mg/mL MTT plus 20% Tween 80 were added and incubated for 3 h. MIC values were defined as the lowest concentration of drug that inhibited 90% of the OD_580_ MTT colour conversion (IC_90_) compared to growth control wells with no drug added.
2. MIC determinations on solid media were performed by the agar dilution method^17^. Briefly, 24-well plates containing MHA with serial two-fold dilutions of tigecycline, colistin and fosfomycin were prepared in duplicates (V_F_ = 1 mL/well). Upon agar solidification, each well was inoculated with 10 µL of a bacterial suspension (ca. 5 × 10^3^ CFU/well) and plates incubated for 18–20 h. Alternatively, plates were prepared as described below (see Semi High-Throughput Synergy Screen) to calculate the sub-inhibitory MICs. The MIC was considered as the lowest value that completely inhibited visible growth.
3. The minimum bactericidal concentration (MBC) was determined to discern bacteriostatic from bactericidal activities. Prior to MTT addition, 10 μL/well were transferred to 96-well LB agar plates and further incubated for 24 h before addition of resazurin (30 μL/well). MBC was defined as the lowest concentration of drug preventing a colour change from blue to pink. A compound was considered bactericidal when MBC/MIC ≤ 4^14^.

### Semi high-throughput synergy screen (sHTSS)

The FDA library (n = 1430, 10 mM) was screened to identify synergistic partners of tigecycline, colistin and fosfomycin (Primary Compounds, PCs) against *K. pneumoniae*^14^. Briefly, an overnight culture of *K. pneumoniae* was diluted to 10^5^ cells/mL in 22 mL of MHB medium containing 0.5% agar (top agar) and uniformly poured over 45 mL of MHB-1.5% agar (bottom agar) in duplicate OmniTrays (Nunc). PCs were added to the bottom agar at sub-inhibitory concentrations (MIC_sub_) of 0.125 and 0.25 mg/L for tigecycline, 0.003 and 0.007 mg/L for colistin and 16 and 32 mg/L for fosfomycin, which corresponded to concentration range between 1/8 × and 1/2 × the MIC values by sHTSS of the compounds alone (Table S2).

FDA-library compounds (0.1 and 1 mM) were transferred from 96-well tester plates onto top agar cell lawns using a pin replicator (1.6 mm pin diameter), which transferred approximately 200 nL/pin (0.2–2 nmol of each compound). OmniTrays were incubated overnight before inhibition zones measurement. Synergy was illustrated by an increase of the inhibition zones in the PC-containing agar plates (Figure S1).

Hits were classified in four categories: (1) synergy (Y): compounds whose inhibition zones increased at the two PCs MIC_sub_ tested (diameter MIC_sub1_ and diameter MIC_sub2_ > diameter_Control_); (2) likely synergy (Y/N): compounds whose inhibition zones increased at only one PC MIC_sub_ (diameter MIC_sub1_ or diameter MIC_sub2_ > diameter_Control_); (3) no interaction (N): compounds with no change in their inhibition zones (diameter MIC_sub1_ and diameter MIC_sub2_ = diameter_Control_); and, (4) likely antagonism (A): compounds with decreased inhibition zones (diameter MIC_sub1_ and diameter MIC_sub2_ < diameter_Control_).

### Secondary validation assays

*Checkerboard assays*. CBA were performed in 96-well plates using freshly prepared CAMHB. Each well was inoculated with 100 µL of 5 × 10^5^ CFU/mL (V_F_ = 200 µL). Pre-inocula were prepared by direct suspension of bacteria grown overnight in MHB and plates incubated for 24 h before determination of the compound activities alone and in combination^14, 18^. Fractional Inhibitory Concentration Indexes (FICI) were calculated as:

1. FICI = FIC_A_ + FIC_B_
2. FIC_A_ = [MIC_A_ in the presence of B]/MIC_A_
3. FIC_B_ = [MIC_B_ in the presence of A]/MIC_B_

Synergy was defined as FICI ≤ 0.5, antagonism as FICI > 4.0, and no interaction when the FICI was between 0.5 and 4.0^20^. Similarly, the Fractional Bactericidal Concentration Index (FBCI) was calculated as above described based on MBC values for each combination using the resazurin method^18^.

*Time-kill assays.* Duplicates of exponentially growing *K. pneumoniae* cultures were inoculated in CAMHB 96-well plates (V_F_ = 280 µL/well; 5 × 10^5^ CFU/mL) containing increasing compound concentrations (0.1×, 0.25×, 1×, 4×, 10× MIC values) of compounds alone or in pairwise or triple combinations. At predefined time points (0, 2, 4, 6, 8, 24 and 48 h), the bacterial population from each well was quantified by spot-platting 10-fold serial dilutions on MHA plates. Plates were incubated overnight, and CFU/mL calculated; the lower limit of detection was 50 CFU/mL. Combo test concentrations were selected based on dose–response curves of the compound alone (typically 0.25× MIC and/or 1× MIC) or up to 300 mg/L in the case of inactive hits. Zidovudine combinations were tested against the 12 strains (including those showing high-level zidovudine resistance) at concentrations up to 1 mg/L to match physiological relevant concentrations; i.e., maximum concentration (C_max_) of zidovudine observed in human plasma after intravenously administration (1.1–1.8 mg/L)^21^. MIC assays were run in parallel with the same inoculum as internal controls of compound activity. Synergistic and bactericidal activities were evaluated after 8, 24 and 48 h of incubation. A synergistic combination was defined as a ≥ 2 log_10_ CFU/mL decrease in the bacterial count of the combination compared to the most active single agent at any time point (8, 24 and 48 h). Antagonism was defined as a ≥ 2 log_10_ increase in CFU/mL between the combination and the most active single agent. All other cases were defined as indifferent. Bactericidal activity was defined as a ≥ 3 log_10_ CFU/mL reduction at any time point (8, 24 and 48 h) compared to the initial inoculum^22^.

### Cytotoxicity assays

The MTT cytotoxicity test^23^ was used to determine the cytotoxicity of the azithromycin- and zidovudine-based combinations in the HepG2 cell line (human liver carcinoma cells from ECACC: cat. N° 85011430). Stock cells were cryopreserved in 90% foetal bovine serum (FBS, Gibco) plus 10% DMSO. Cells were thawed from stocks and cultured in flasks with pre-warmed (37 °C) Dulbecco's Modified Eagle's Medium (DMEM) (Gibco) supplemented with 10% FBS, 1% Glutamax and 100 µg/mL streptomycin, penicillin and ciprofloxacin for 24 h in a controlled 5% CO_2_ atmosphere at 37 °C. Two passages were performed before the cytotoxicity assays. After microscopic evaluation of a semi-confluent monolayer, cells were removed by enzymatic digestion with 0.05% trypsin–EDTA solution (5 min at 37 °C) and then neutralized with supplemented DMEM. Cells were centrifuged (240*g*, 5 min) and the medium replaced to adjust the cell suspension to 10^6^ cells per plate. Cells were seeded in 96-well flat bottom plates transferring 100 µL per well (2 × 10^4^ cells) and incubated under conditions described above. After 24 h, media was aspirated and replaced by fresh DMEM supplemented with 10% FBS, 1% Glutamax and containing the compounds dissolved. Compounds were added both alone and in combination at the same concentrations used in the TKA. Growth controls and blanks with medium were also included in the plate layout. Cells were incubated for additional 24 h and then, 10 µL of MTT dissolved in PBS (5 mg/mL) added to each well. Plates were further incubated for 2 h before the MTT solution was removed and MTT crystals solubilized in 100 µL/well of DMSO, mixed and the absorbance measured (OD_570_ and OD_650_). The percentage of cell viability was determined based on the untreated controls. Treatments were considered cytotoxic when cell viability was reduced to < 70% of the growth controls, according to the international standard protocols^23^. Experiments were performed in technical triplicates and biological replicates. Graphical representation and statistical analysis to compare each concentration were performed by one-way ANOVA test in GraphPad Prism software (version 8.0.2).

## Results

### Synergy screens of the FDA library in combination with last-line antibiotics identified novel combinations against *K. pneumoniae*

The MIC of the three PCs (colistin, fosfomycin and tigecycline) was determined in liquid and solid media; MIC values were also determined under the same conditions used in the sHTSS. By this method, we observed a 100-fold higher activity for colistin compared to its activity in both liquid and solid media (Table S2). This could be explained by differences in the drug distribution or growth state of the bacteria, since in the sHTSS method cells are embedded in soft agar instead of growing on the agar surface.

**Figure 1:**
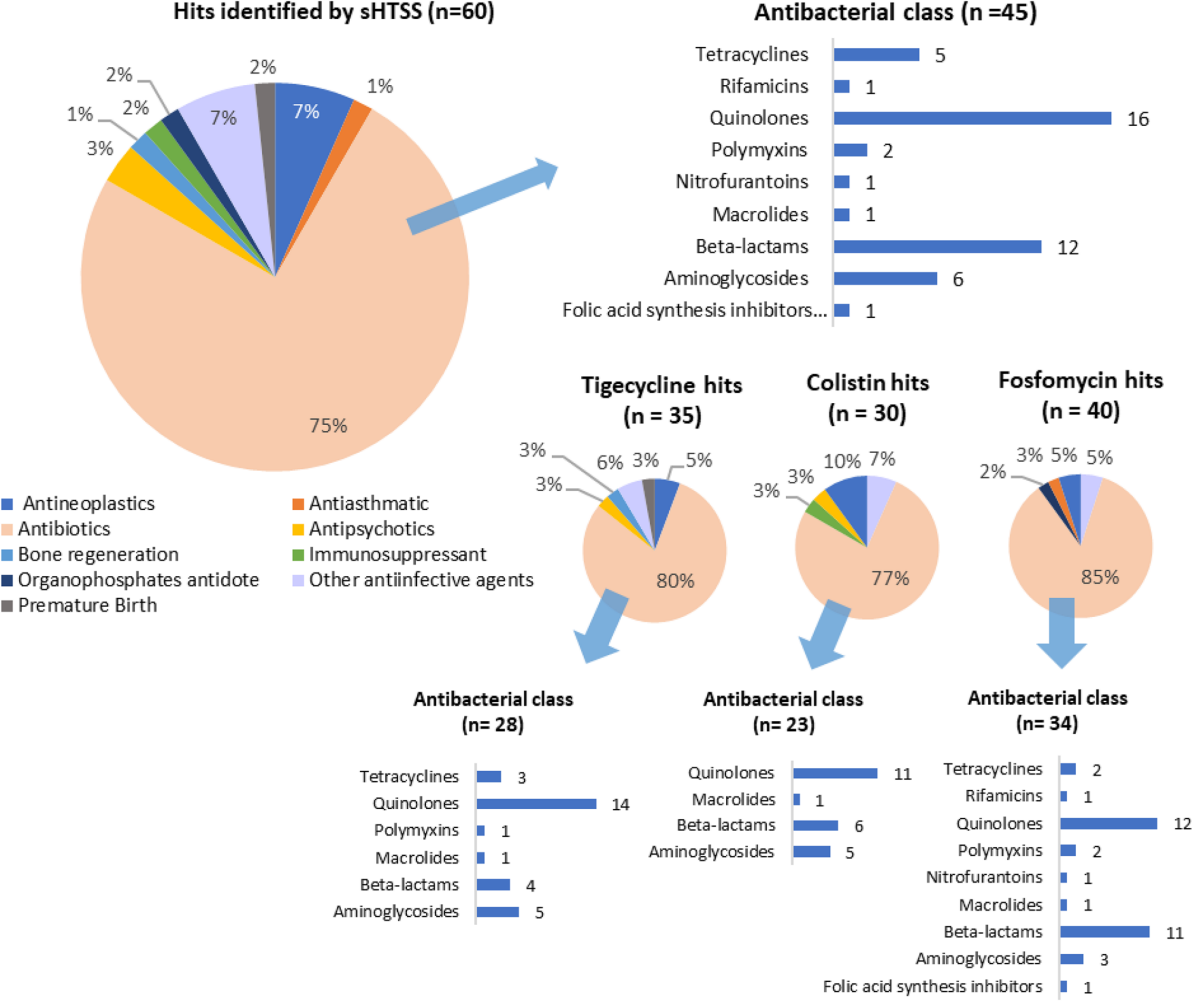
FDA compounds with favourable interactions with tigecycline, colistin and fosfomycin identified by sHTSS and classified by their therapeutic use. Other anti-infective agents include anti-parasitic, antiseptic and antiviral agents. Duplicate hits were removed from analysis. *sHTSS* semi-high throughput synergy screen.

Overall, 109 FDA compounds enhanced the activity of the PCs with hit discovery rates of 2.59% (n = 37), 2.17% (n = 31), and 2.87% (n = 41) for tigecycline, colistin and fosfomycin, respectively. According to the interaction ranking criteria (see “Materials and methods”), 19, 9 and 18 interactions were classified as synergistic, and 18, 22 and 23 as likely synergistic with tigecycline, colistin and fosfomycin, respectively. Additionally, 6, 10 and 12 interactions were classified as likely antagonistic with tigecycline, colistin and fosfomycin, respectively. We found some promiscuous compounds able to enhance the activity of more than one PC; thereby, among the 109 compounds that initially enhanced the activity of any of the PCs, there were 60 unique hits. Classification by their therapeutic use revealed most hits were known antibacterial agents (75%), including quinolones (n = 16), β-lactams (n = 12) and aminoglycosides (n = 6), with non-antibacterial compounds (n = 15) including other anti-infective agents (7%), antineoplastics (7%) or antipsychotics (3%), among others. Percentages were similar among the three PCs (Fig. 1). A comprehensive hit list, inhibition zones and correlation with secondary validation assays are displayed in Table S3.

### Checkerboard assay displayed low validation rates

Fifty-three sHTSS interactions classified as synergy (n = 50) or antagonism (n = 3) for any of the PCs were evaluated by CBA. FICI and FBCI indexes were calculated based on their MIC and MBC values, respectively (Table S4). Interactions were validated in 8 out of the 53 (15.09%) combinations. However, only those of colistin showed a certain degree of validation (7 out of 12 combinations tested, 58.33%), while no validation was observed with tigecycline (0 out of 16) or fosfomycin (1 out of 25) (Table S5).

### Time-kill studies revealed novel promising pairwise combinations against *K. pneumoniae*

**Figure 2:**
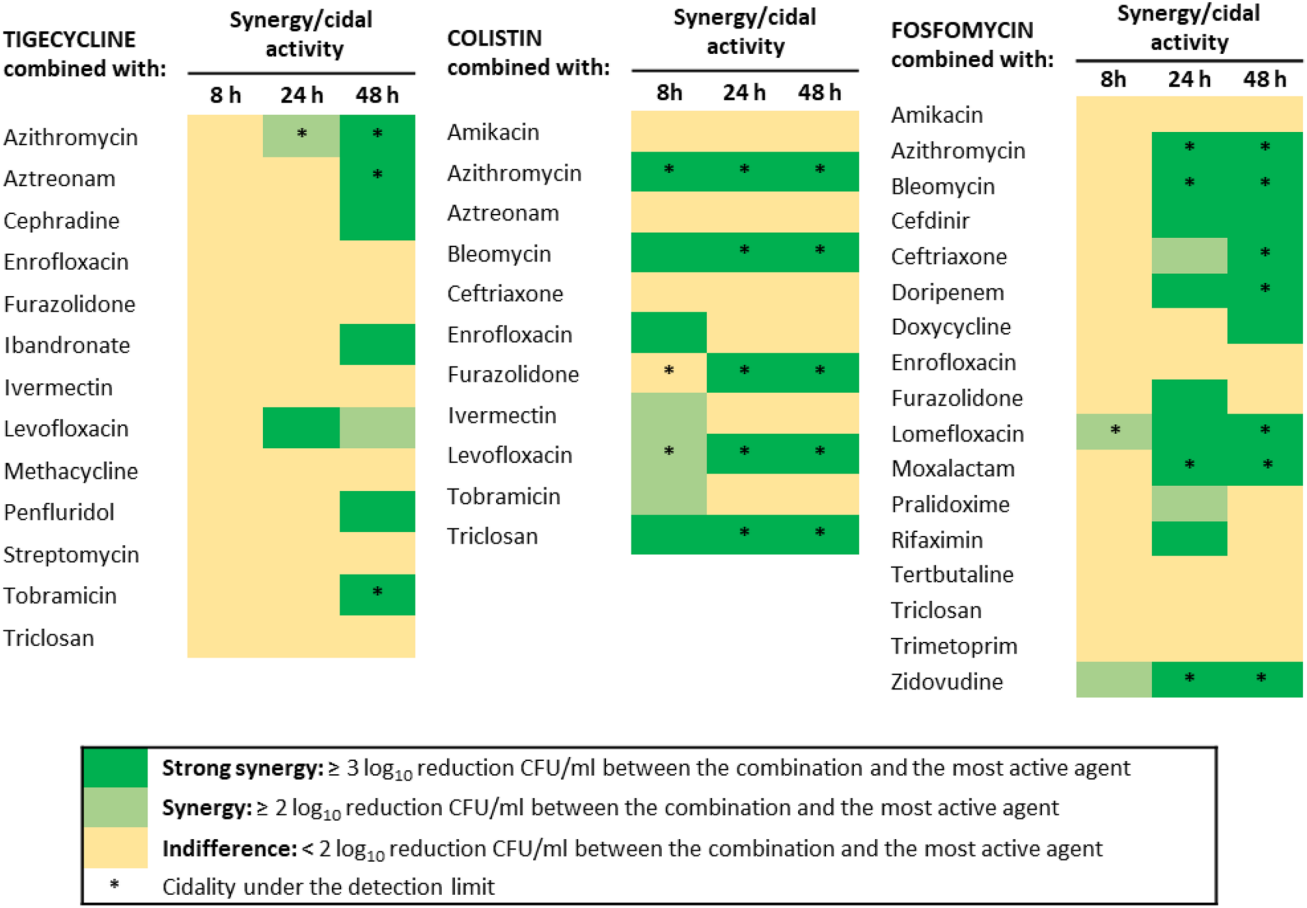
TKA drug interaction map of sHTSS hits. Forty-one sHTSS hits were selected for secondary validation TKA based on pharmacological and clinical translation potential assessments. Drug interaction was assessed after 8, 24 and 48 h. The degree of interaction was determined as the log_10_CFU/mL increased reduction between the combo and the most active agent alone (see figure inset). Combinations that kill down to the limit of detection were marked by an asterisk.

**Figure 3:**
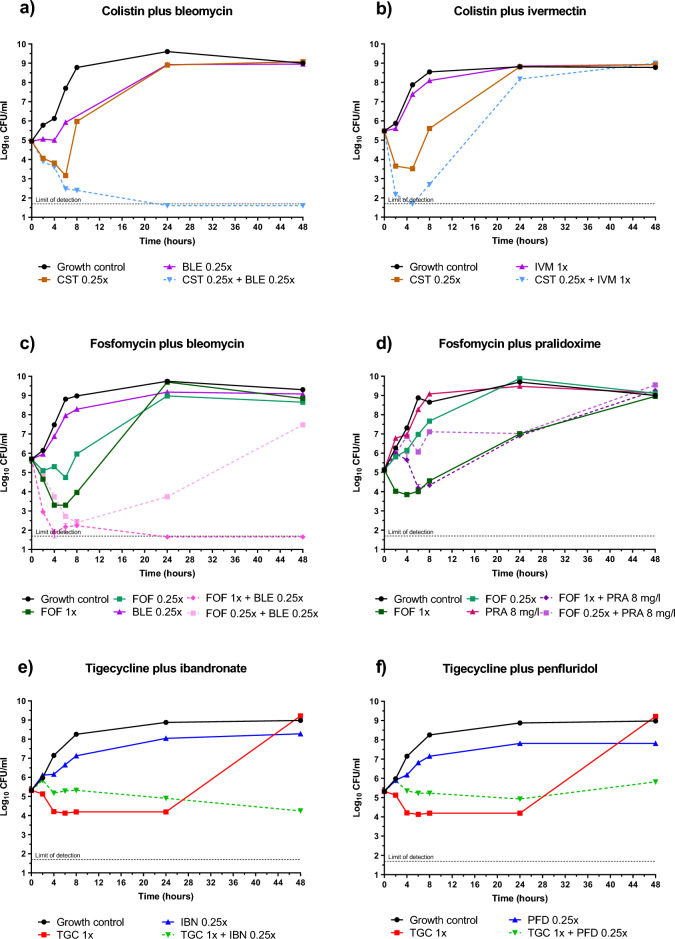
Novel combinations of non-antibiotic drugs with the PCs against *K. pneumoniae* ATCC 13883. (**a**,**b**) Colistin combined with bleomycin or ivermectin. Bleomycin strongly enhanced colistin to the limit of detection; ivermectin showed synergy up to 8 h of incubation with colistin, after this time point a rebound was observed, similarly to both compounds alone; (**c**,**d**) Fosfomycin combined with bleomycin (bactericidal from early hours) or pralidoxime showed synergy with fosfomycin at 8 h, followed by a rebound (**e**,**f**) Tigecycline combined with ibandronate or penfluridol showed static activity and synergy at 48 h. *BLE* bleomycin, *CST* colistin, *FOF* fosfomycin, *IBN* ibandronate, *IVM* ivermectin, *PFD* penfluridol, *PRA* pralidoxime, *TGC* tigecycline, *MIC*_*BLE*_ = 0.25 mg/L; MIC_CST_ = 1 mg/L; MIC_FOF_ ≥ 128 mg/L; MIC_IVM_ > 64 mg/L (assumed in 64 mg/L); MIC_TGC_ = 0.5 mg/L; MIC_IBN_, MIC_PFD_ and MIC_PRA_ > 32 mg/L (all assumed in 32 mg/L).

Forty-one combinations (35 classified as “Y” or “Y/N” and 6 as “A”, see “Materials and methods”) were studied by TKA to assess their pharmacological and clinical translation potential (Fig. 2; Table S6). Overall, TKA showed a synergy validation rate of 65.85% (27 out of 41 combinations) at any of the predefined time points. Specific confirmation rates for each PC were 72.72% (8/11) for colistin, 70.58% (12/17) for fosfomycin, and 53.84% (7/13) for tigecycline (Table S5). Effective combinations were identified with antimicrobials such as azithromycin (displaying potent synergistic and bactericidal activities with all three PCs). Levofloxacin, furazolidone and the antiseptic triclosan were specific of colistin, while fosfomycin combinations with cefdinir, ceftriaxone, doripenem, lomefloxacin and moxalactam showed potent synergistic interactions at the later time points, reaching the limit of detection. Tigecycline showed some degree of interaction with aztreonam, tobramycin, cephradine, or levofloxacin (Fig. 2). Interestingly, novel synergistic interactions with non-antibiotic drugs were confirmed in these studies, including colistin plus the antineoplastic bleomycin or the antiparasitic ivermectin, fosfomycin plus bleomycin, pralidoxime (a poisoning antidote) or zidovudine^24^, and tigecycline combined with bisphosphonate ibandronate or the antipsychotic penfluridol (Fig. 3).

### Novel zidovudine- and azithromycin-based combinations are effective against MDR/XDR *K. pneumoniae* strains

Zidovudine and azithromycin, identified in the sHTSS as strong enhancers of colistin, fosfomycin and tigecycline, were selected for further investigation in the treatment of infections caused by MDR *K. pneumoniae.*

First, MIC determinations were performed against a panel of 12 MDR/XDR *K. pneumoniae* strains. MIC values of zidovudine (MIC_ZDV_) ranged from 0.25 to ≥ 64 mg/L, with nine strains showing low MIC_ZDV_ values (0.25–2 mg/L) and three MIC values ≥ 16 mg/L; in consequence, a concentration of 2 mg/L was used in this study as a possible cut-off for zidovudine resistance. MIC values of azithromycin (MIC_AZM_) ranged from 4 to ≥ 64 mg/L, with ten strains showing MIC_AZM_ values lower than 8 mg/L and only two with MIC values ≥ 64 mg/L. There are no CLSI or EUCAST guidelines describing azithromycin clinical breakpoints for enterobacteria, except for *Salmonella* Typhi and *Shigella* spp.^25^. However, in our experiments, azithromycin exhibited a clear cut-off at 8–16 mg/L, which were in the same range of values as those epidemiological cut-offs (ECOFFs) stablished by EUCAST for azithromycin in other enterobacteria^26^. The activity of zidovudine and azithromycin was thus in the same concentration range (low mg/L) of other currently used last-line antibiotics (Table S7).

**Figure 4:**
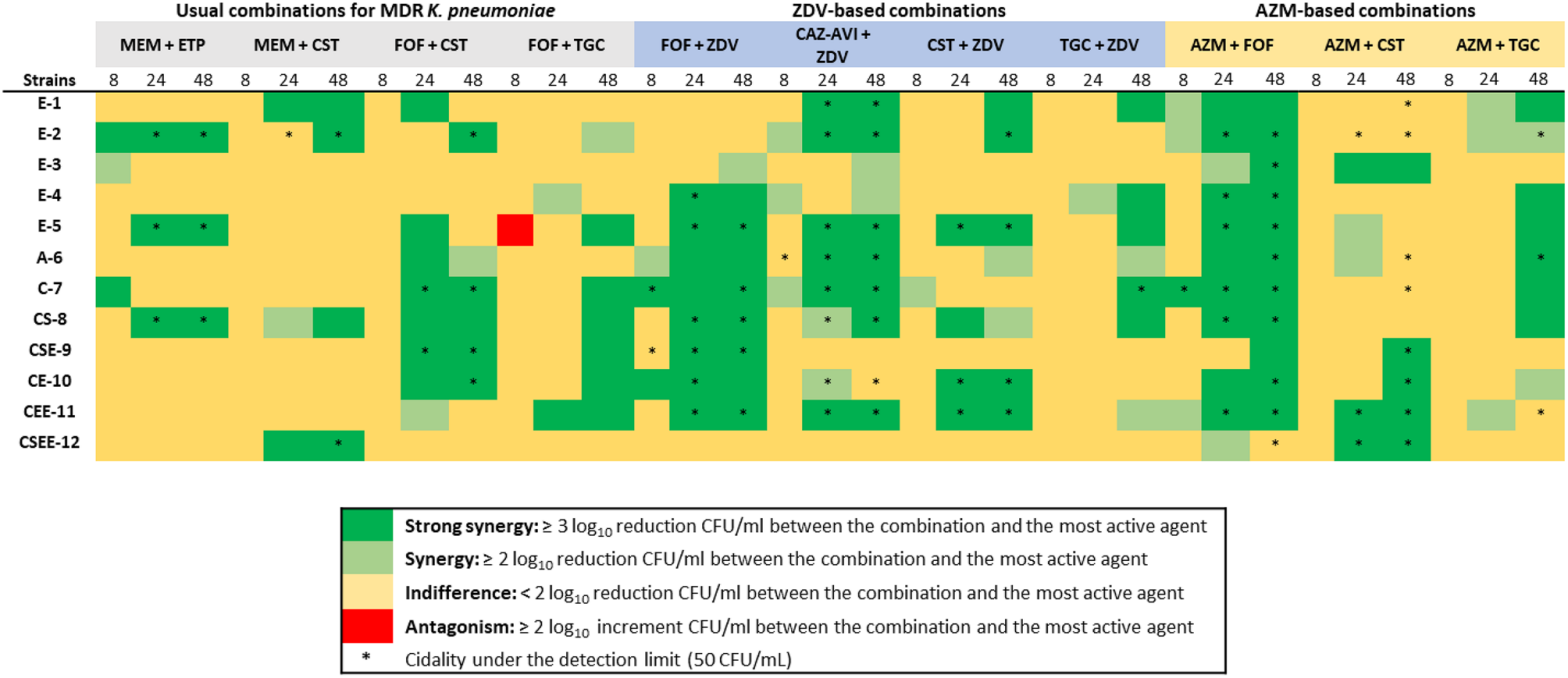
TKA heat map representation of the synergistic and bactericidal activities of clinically used versus novel combinations against *K. pneumoniae* strains. ZDV-based combinations were tested at concentrations ≤ 1 mg/L, reflecting physiologically achieved concentrations. The most favourable outcome is displayed when several concentrations were tested for the same drug. *CAZ-AVI* ceftazidime-avibactam, *CST* colistin, *ETP* ertapenem, *FOF* fosfomycin, *MEM* meropenem, *TGC* tigecycline, *ZDV* zidovudine, *AZM* azithromycin. Data supporting this summary figure is displayed in Figures S2, S3 and S4.

Then, zidovudine- and azithromycin-based pairwise combinations were tested against the same panel of strains. In order to understand the potential translational impact of these combinations and provide a temporal component to the interaction analysis, we compared their activities with those of the current clinically used last-resort combinations against infections caused by MDR/XDR *K. pneumoniae* by time-kill assays (Fig. 4)*.* At any time-point (8, 24 and 48 h), positive interactions and bactericidal profiles among currently used combinations for MDR treatment were observed in five and three strains for meropenem/ertapenem (41.6% and 25%), four and two strains for meropenem/colistin (33.33% and 16.6%), nine and four strains for fosfomycin/colistin (75% and 33.3%) and eight and none strains for fosfomycin/tigecycline (66.6% and 0%), respectively (Fig. 4, Figure S2). In stark contrast, a high number of synergistic interactions were obtained with zidovudine and azithromycin-based combinations across all strains.

For the zidovudine combinations, ceftazidime-avibactam plus zidovudine was the most active among all strains, showing a positive interaction in 10 out of 12 (83.3%). In eight of the strains (66.6%) the killing activity was below the limit of detection after 24 h of incubation, preventing bacterial regrowth (a proxy for sterilizing activity). The combination of fosfomycin plus zidovudine was synergistic in 9 out of 12 strains (75%), showing a potent and rapid initial decrease in bacterial counts after 8 h in four strains and bactericidal activity down to the limit of detection in six strains. The combination of zidovudine plus colistin was synergistic in 9 out of 12 (75%) strains with killing activity to the limit of detection in four (33.3%). Finally, zidovudine plus tigecycline was the less potent with late synergy (48 h) in 7 out of 12 strains (58.3%), being bactericidal only against the C-7 strain (Fig. 4, Figure S3).

For the azithromycin combinations, azithromycin plus fosfomycin was the most potent, showing positive interactions at any time point in all the strains tested and bactericidal in 10 out of the 12 strains (83.3%). The potency of this combination was evident when compared to the activity of the drugs alone; neither showed long-lasting bactericidal activity, with a static effect or no activity (azithromycin), and rapid bactericidal activity followed by bacterial regrowth from the 8-h time-point (fosfomycin). In addition, in most strains combined bactericidal effects were already detected at early time points (4–8 h). The combination azithromycin/colistin was synergistic at any time point in 7 out of 12 (58.3%) and bactericidal in 8 out of the 12 strains (66.6%), including two colistin-resistant strains that had viable counts below the limit of detection (50 CFU/mL). The combination azithromycin/tigecycline was synergistic at any time point against 9 out the 12 (75%) strains, being bactericidal in three strains (E-2, A-6 and CEE-11) (Fig. 4, Figure S4).

**Figure 5:**
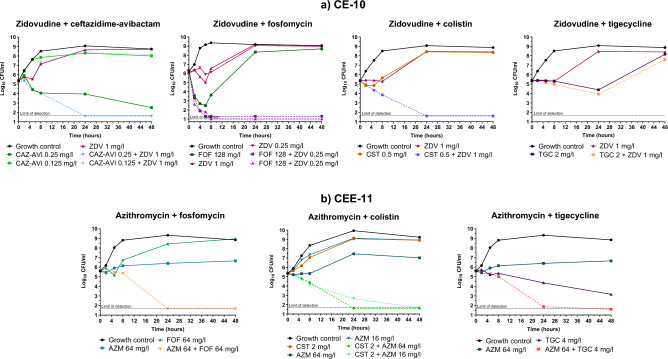
Zidovudine- and azithromycin-based combinations are active against *K. pneumoniae* strains resistant to zidovudine and azithromycin. (**a**) Zidovudine enhanced the activities of ceftazidime-avibactam, fosfomycin and colistin even at low sub-inhibitory concentrations (0.004–0.015xMIC_ZDV_), against CE-10 (*bla*_OXA-48_ + *bla*_CTX-M15_). MIC_CAZ-AVI_ = 0.25 mg/L, MIC_CST_ = 1 mg/L, MIC_FOF_ = 64 mg/L, MIC_TGC_ = 1 mg/L, MIC_ZDV_ = 64 mg/L; (**b**) azithromycin enhanced the activities of fosfomycin, colistin and tigecycline at subinhibitory concentration (0.25–1xMIC_AZM_) against CEE-11 (*bla*_KPC-3_ + *bla*_SHV-1_ + *bla*_TEM-1_). MIC_AZM_ ≥ 64 mg/L, MIC_CST_ = 2 mg/L, MIC_FOF_ > 64 mg/L, MIC_TGC_ = 4 mg/L. *CAZ-AVI* ceftazidime-avibactam, *CST* colistin, *FOF* fosfomycin, *TGC* tigecycline, *ZDV* zidovudine, *AZM* azithromycin.

The strong interaction observed in the zidovudine- and azithromycin-based combinations was maintained even in those strains (CE-10 and CEE-11) with high MIC values for both compounds. Even at concentrations at which zidovudine or azithromycin had minor antibacterial effects, the combinations (except zidovudine/tigecycline) were synergistic and highly bactericidal, indicating that these treatments can overcome resistance (Fig. 5).

**Figure 6:**
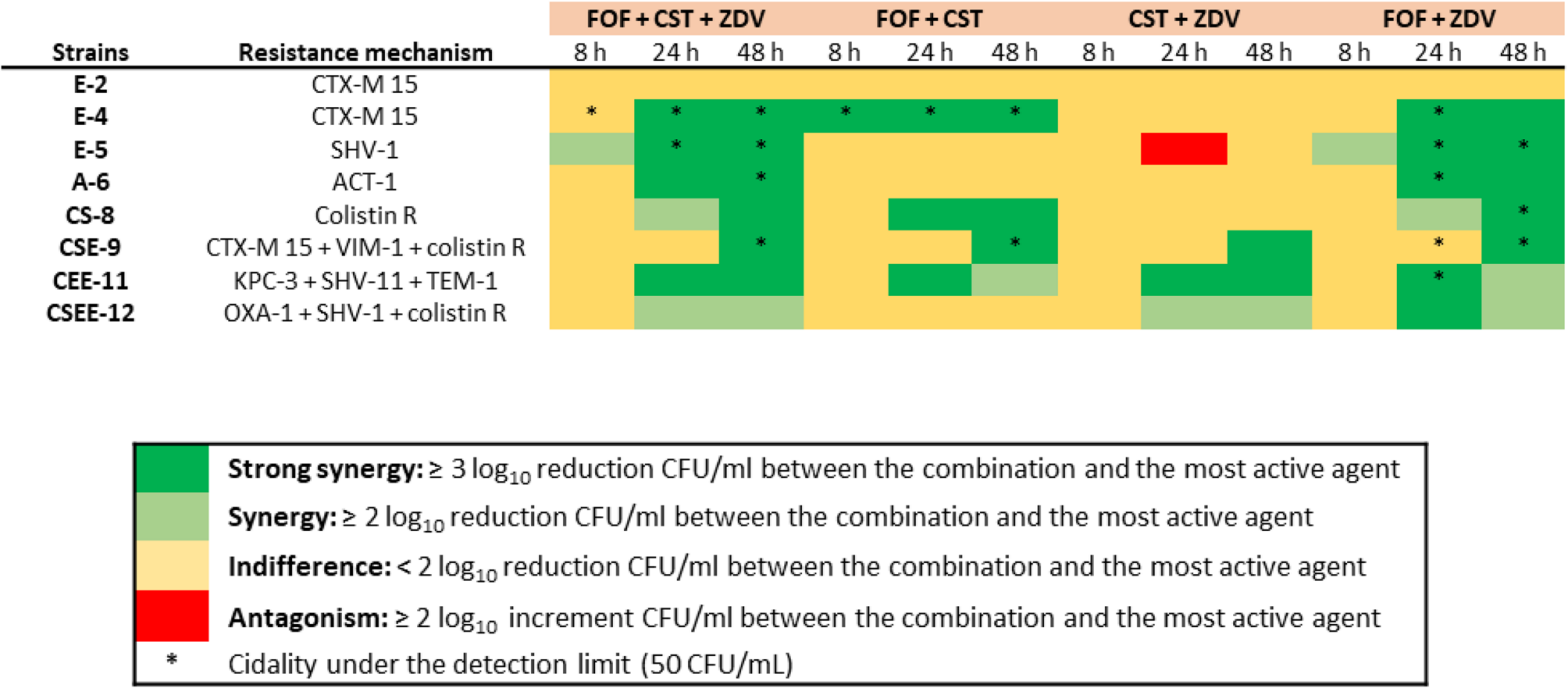
TKA heat map representation of the synergistic and bactericidal activities of the triple fosfomycin/colistin/zidovudine combination compared to the pairwise combination against eight *K. pneumoniae* strains. Combo tested concentrations at 0.25–1 × MIC. Data supporting this summary figure is displayed in Figure S5. *CST* colistin, *FOF* fosfomycin, *ZDV* zidovudine.

Finally, we explored whether triple combinations of synergistic pairwise combos would be more potent that the combination of the two drugs. We showed that, while carbapenem-based combinations had little synergistic interaction profiles, the combination of two last-line antibiotics (fosfomycin/colistin) had an overall synergy rate of 75% and that zidovudine displayed strong synergy with both drugs (Fig. 4). We thus aimed to characterise whether the addition of zidovudine to the fosfomycin/colistin combination could further potentiate their synergistic interactions, as previously described^14, 27, 28^. The triple combination was highly active showing positive interactions in 7 out of the 8 (87.5%) strains tested and bactericidal activities in 4 out of 8 (50%). The triple combination was superior to the colistin/zidovudine and fosfomycin/colistin pairwise combos but added little or no benefit compared to the fosfomycin/zidovudine combo (Fig. 6).

### Zidovudine- and azithromycin-based combos are not cytotoxic

**Figure 7:**
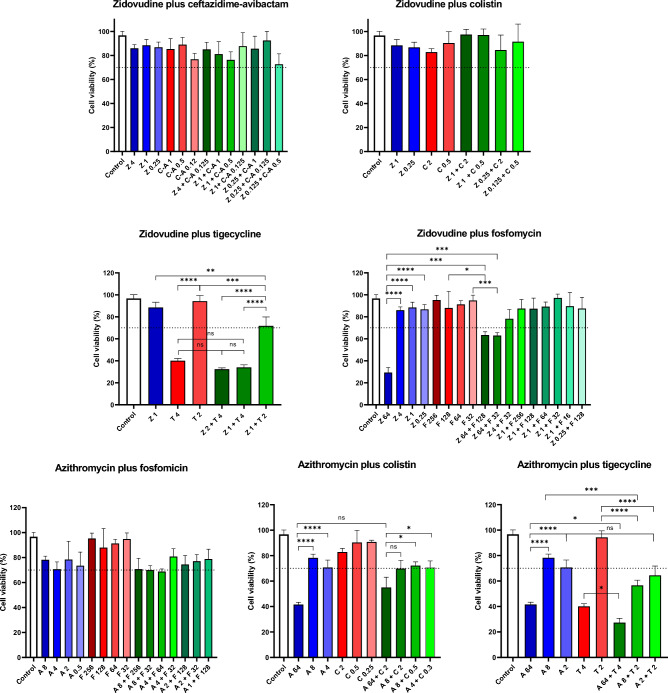
Cytotoxicity of zidovudine- and azithromycin-based combinations in a HepG2 cell line. Concentration of compounds are expressed in mg/L. Cell suspensions with no compounds added were used as positive control. Statistical differences are displayed by asterisks; *p ≤ 0.05; **p ≤ 0.01; ***p ≤ 0.001; ****p ≤ 0.0001; ns, *p* > 0.05; *A* azithromycin, *C-A* ceftazidime-avibactam, *C* colistin, *F* fosfomycin, *T* tigecycline, *Z* zidovudine.

MTT cytotoxicity assays in a HepG2 cell line were performed to evaluate drugs alone and in combination at those concentrations that showed bactericidal activities by TKA. Cell viability was > 70% (which is considered non-cytotoxic according to standard international protocols^23^) for most drugs and combinations tested. Only tigecycline, out of the three last-line antibiotics, was cytotoxic at 4 mg/L (40% cell viability), but not at 2 mg/L (> 90% cell viability), which is near to the mean C_max_ described after standard intravenous dose of 100 mg of tigecycline (1.45 mg/L)^29^. Zidovudine and azithromycin were cytotoxic at the highest concentration tested (64 mg/L) (< 40% cell viability), but not at any of the lowest concentrations used in the combinations. The combination might be even beneficial since zidovudine toxicity was reduced in the case of zidovudine plus fosfomycin. Importantly, no combination was more cytotoxic than the drugs alone in the same combination (Fig. 7).

## Discussion

**Figure 8:**
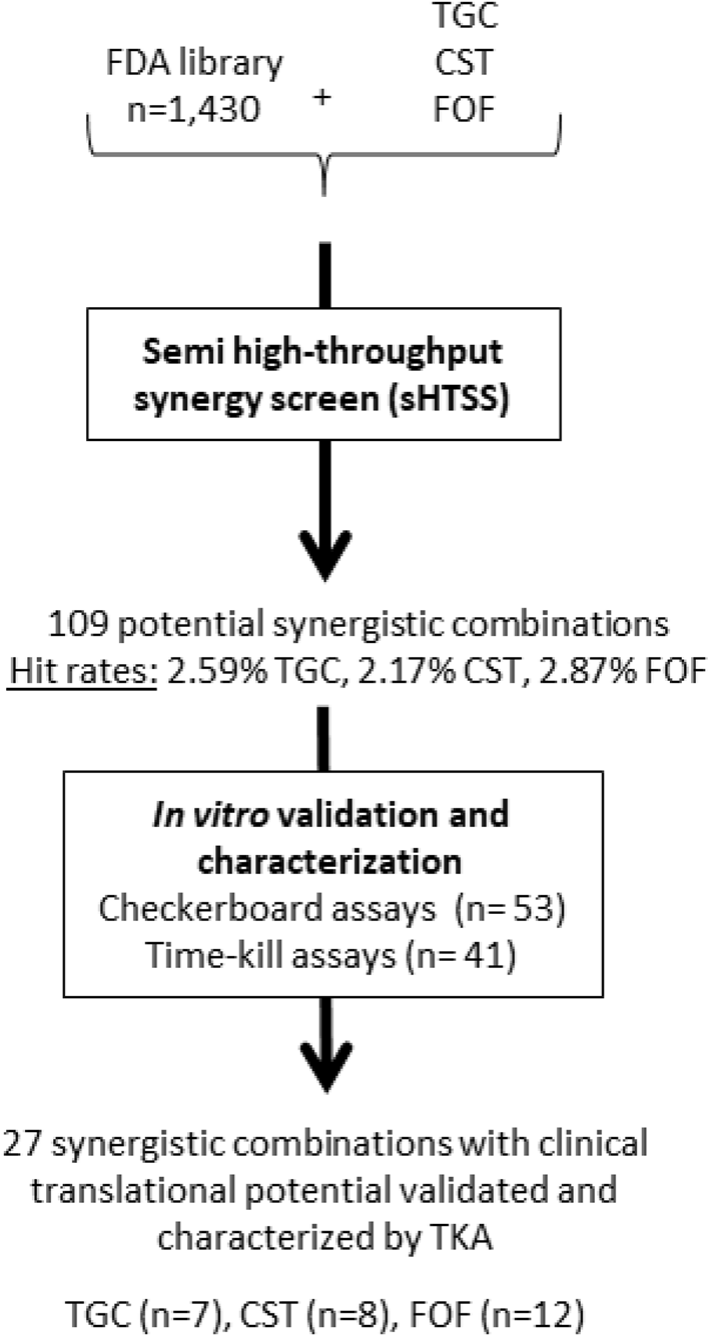
Synergy screen progression cascade. Screening activities were performed with *K. pneumoniae* ATCC 13883. The number of compounds tested, hit rates and hits validated are indicated at every step. *TGC* tigecycline, *CST* colistin, *FOF* fosfomycin, *TKA* time-kill assay.

sHTSS were developed for mycobacteria and successfully identified novel combinations enhancing the activity of known antimicrobials^14, 28^. Here, we adapted the sHTSS methodology to find active combinations against *K. pneumoniae*. sHTSS allowed for rapid and clear drug interaction readouts derived from inhibition zones in agar media. A general overview of the screening and validation progression cascade is displayed in Fig. 8, with Table S5 displaying a more comprehensive summary.

Our screens in *K. pneumoniae* yielded hit rates ranging from 2.17 to 2.87%, higher than that observed in *M. smegmatis* (1.4%)^14^, but similar to other studies against Gram-negative bacilli with comparable hit rates (1.87%, tigecycline/5.54%, colistin)^30^. A large proportion were known antimicrobial drugs (82%), including antibacterial (75%) and other anti-infective agents (7%) (Fig. 1). The overrepresentation of antimicrobials in synergy screening programs enhancing the activity of other antimicrobials is similarly described in other studies with *K. pneumoniae*^30, 31^ and *M. smegmatis*^14^. Hind et al*.* observed this finding specially associated with *K. pneumoniae*, while they identified more heterogeneous targets with other Gram-negative bacteria^30^. Secondary validation assays performed by CBA and TKA provided variable confirmation rates; CBA yielded low sHTSS confirmation rates (15.09%), especially in the case of tigecycline and fosfomycin, while these increased to 65.85% when tested by TKA (Table S5).

While performing CBA, we also measured the MBC (a fixed time point bactericidal parameter) to also determine the FBCI. This index has been largely disregarded in synergy studies, but some authors suggested FBCI might be a better predictor of drug interaction than FICI^32–34^. As an example, Figure S6 shows validation results of the tigecycline/aztreonam combination; a clear “no interaction” profile was found by CBA (FICI = 1), while a tendency towards synergism was observed by FBCI (FBCI = 0.75). The use of a fixed time point bactericidal parameter was able to predict TKA data, i.e., the combination showed no synergy compared to the most active drug alone at 24 h (although with a tendency to reduce the bacterial count compared to tigecycline alone) but prevented growth rebound at 48 h, indicating a strong synergistic interaction.

The fixed time point limitation of the MBC assays is addressed in TKA by the inclusion of longitudinal pharmacodynamic data, allowing for a more robust methodology to characterise drug interaction dynamics, a paradigm shift in antimicrobial development methodologies^35^. We decided to perform extended TKA up to 48 h of incubation with the aim to prioritise the most effective combinations that maintained bactericidal activities up to the end of the assay. Moreover, the 48 h time point allow us to identify combinations that could potentially lead to therapeutic failure when used in the clinical practice, i.e., combinations considered initially effective but that rebounded after the 24 h time point, which could be a better proxy of sterilization and, thus, potency of the combination.

The highest number of validated combinations was obtained for colistin and fosfomycin, antibiotics targeting the outer membrane and cell wall, respectively. Both antibiotics have been associated with enhance killing when in combination with intracellular targeting compounds, explained by an increase in permeability^30, 36, 37^. In agreement with our study, synergistic interactions with the three PCs have been reported for the treatment of MDR enterobacteria^38, 39^. In fact, synergy was demonstrated between tigecycline and aminoglycosides^40–42^, colistin and levofloxacin^43, 44^, fosfomycin and cephalosporins^37^ or doripenem^45, 46^ against *K. pneumoniae*. A recent review on in vitro fosfomycin combinations supports our findings in which we did not observe synergy between fosfomycin and amikacin or trimethoprim^47^. Using CBA, Ontong et al*.* reported synergy between colistin/amikacin and colistin/tobramycin for 72.72% and 45.45% of MDR *K. pneumoniae* strains, respectively^43^. We also observed synergy by CBA for the latter combination, but such interactions were not confirmed by TKA (Tables S3, S6). This highlights again TKA as a much better proxy than CBA to validate synergistic combinations.

Our synergy screen progression cascade validated several novel combinations with non-antibiotics: (1)* Tigecycline plus ibandronate or penfluridol.* Ibandronate and other bisphosphonate derivatives are anti-parasitic drugs inhibiting the synthesis of essential isoprenoids^48, 49^. The antipsychotic penfluridol displayed partial synergistic activity with aminoglycosides and β-lactams against *Enterococcus faecalis*^50^. To the best of our knowledge, here we report for the first time the antibacterial activity of both drugs with tigecycline against enterobacteria; (2)* Bleomycin plus colistin or fosfomycin.* Although the cytotoxicity of bleomycin poses a barrier to an antibacterial repurposing approach, our results could set up the basis for the development of analogues or dosage-based studies to minimise its toxicity.

Out of all the hits identified in our screening, based on their translational potential under a repurposing strategy, we decided to focus on zidovudine and azithromycin and performed in vitro combinatorial TKA against an extended panel of MDR *K. pneumoniae* strains and compare their activity against combinations already used in the clinical setting to treat this type of infections (Fig. 4). For this, we considered, not only the increased bactericidal activity of the combination compared to the drugs alone, but also the ability of the combination to completely eradicate bacteria down to the limit of detection of the assay (a proxy for sterilization of the culture). Our TKA data showed high rates of synergistic and killing activities of novel combinations even against strains with concurrent resistance mechanisms to these and other antimicrobials (Fig. 5), with no increased cytotoxicity (Fig. 7).

Zidovudine was the first commercial antiretroviral agent for HIV/AIDS treatment. Since 1980s, its antibacterial activity against enterobacteria is attributed to its targeting of bacterial thymidine kinases^51^. However, concerns about toxicity and the emergence of resistant strains have limited the development of zidovudine-based antibacterial therapies. Synergistic combination therapy could be an option to limit zidovudine toxicity. Several in vitro and/or in vivo studies demonstrated effective antibacterial activities of zidovudine in combination with antibiotics such as fluoroquinolones, trimethoprim, aminoglycosides, tigecycline and polymyxins^51–56^. In a recent report, Antonello et al*.*^24^ described the in vitro and in vivo (*Galleria mellonella* infection model) activity of zidovudine in combination with fosfomycin. We also identified and validated a strong synergistic interaction between zidovudine and fosfomycin where zidovudine was able to restore the activity of fosfomycin, preventing bacterial regrowth (Figure S3), thus confirming their results.

With the aim to explore the role of zidovudine as a general enhancer of last-line antibiotics in the treatment of MDR *K. pneumoniae* infections, we tested zidovudine in combination with other antibiotics against our panel. The strongest interaction was observed with ceftazidime-avibactam, followed by fosfomycin, colistin and tigecycline to a lesser extent (Fig. 4). Safety and efficacy of ceftazidime-avibactam against MDR enterobacteria facilitated its inclusion as first-line therapeutic option for infections caused by CPE. It is administered in monotherapy against OXA-48 (class D) and KPC (class A) producers or associated to aztreonam against class B enzymes (β-lactamases refractory to inhibition by avibactam)^57^. Although the potential for resistance selection appears to be low^58^, the extensive use of ceftazidime-avibactam as a savage therapy will contribute to the emergence of resistance. In fact, resistance linked to mutations in plasmid-borne KPC-3 were reported during ceftazidime-avibactam treatment^59, 60^, and development of resistance to ceftazidime-avibactam is more likely after previous exposure with meropenem-vaborbactam^61, 62^. To the best of our knowledge, this is the first time that the zidovudine/ceftazidime-avibactam combination is evaluated against MDR *K. pneumoniae.* Our promising results might lay the foundations of further studies to support a potential clinical implementation.

The combination of zidovudine plus tigecycline was the least effective one in terms of drug interaction (Figure S3). Nevertheless, TKA were able to reveal important interactions confronting the data generated by CBA^55^*.* The fact that zidovudine/tigecycline showed a lower degree of interaction could be explained because both drugs have intracellular targets. On the contrary, fosfomycin, ceftazidime-avibactam and colistin target the cell wall. One can speculate that the molecular bases underlying this synergism is due to an increased permeability of zidovudine due to the cell wall destabilization properties of the companion drugs, thus increasing its intracellular availability and having a higher effective concentration against the target resulting in a higher effectivity^47, 53, 56, 63^. Adding to this hypothesis, the use of glucose-6-phosphate (added in in vitro experiments to mimic physiological conditions and promote intracellular transport of fosfomycin) might also facilitate zidovudine entrance to the bacterial cell^24^. This could also explain the fact that a triple zidovudine/fosfomycin/colistin combination is not superior to a double zidovudine/fosfomycin combination (Fig. 6). Fosfomycin by itself could provide enough increased permeability to zidovudine to saturate the intracellular target and the addition of colistin might not provide an additional advantage. Thus, in a hypothetical clinical therapy, it would be advisable to treat with only two drugs to limit the toxicity associated with colistin treatment.

For HIV treatment, zidovudine is dosed at 500–600 mg daily oral and 1.5 mg/kg/6 h intravenously. After standard dosage regimens, C_max_ ranging from 1.1 to 1.8 mg/L are achieved^21, 64^. Major toxicities (anemia and neutropenia) are more frequently described at high doses (1.200–1.500 mg/day) after more than 4 weeks of treatment^65, 66^. A few case reports described oral zidovudine overdoses up to 36 g/daily without abnormalities or with slight and transient side-effects such as lethargy^67^. Zidovudine toxicity is associated to the dose, disease stage and prolonged therapy. However, safety profiles observed in HIV-patients together with a short plasma half-life (1.1–2.3 h)^21, 64, 68^ suggest that appearance of zidovudine toxicities are unlikely and previous in vitro and in vivo studies suggested that clinically achievable zidovudine concentrations could be effective against MDR enterobacteria when in combination therapy^24, 56, 63^. In our study, the MIC of zidovudine ranged from 0.25 to ≥ 64 mg/L, which are in accordance with similar studies^24, 53, 56, 63^. We observed potent bactericidal activities of the combinations against most strains at zidovudine concentrations below 1 mg/L (Figure S3), which were non-cytotoxic (Fig. 7). Pharmacokinetics and safety of zidovudine plus colistin combination antimicrobial therapy was evaluated in a clinical trial^69, 70^ and doses of both zidovudine and colistin could be reduced while retaining their therapeutic efficacy^56^. Our data thus suggests that current zidovudine dosing strategies might suffice to treat bacterial infections in humans and that zidovudine associated side effects are unlikely to occur during short-term regimens, as in the context of acute bacterial infections. In addition, zidovudine reduces transmission of ESBL and carbapenemase-containing plasmids, hence supporting zidovudine use in the prevention of the spread of resistant enterobacteria^71^.

Azithromycin is a broad-spectrum macrolide widely prescribed for several indications such as respiratory, genitourinary and dermal infections^72, 73^, with beneficial anti-inflammatory and immunomodulatory properties in critically ill patients^74^ and chronic respiratory disorders^75–78^. Monotherapy use of macrolides has been disregarded in the treatment of severe infections caused by Gram-negative bacteria due to different existing mechanisms of resistance to azithromycin in enterobacteria and the low permeability of their outer membrane^79^. However, the enhanced basicity of azithromycin favours the intracellular uptake in Gram-negative bacteria increasing its efficacy and it is currently used for the treatment of enteric infections such as typhoid^73^. Our progression cascade validated the interaction of azithromycin with all three last-line antibiotics. This was in agreement with several studies reporting the bactericidal and antibiofilm action of azithromycin combined with tigecycline^80^, and colistin^81, 82^ against different Gram-negative bacilli. The interaction of azithromycin with fosfomycin against *K. pneumoniae* is novel, although synergy has been described with erythromycin^83^. This combination was previously assessed in two in vitro studies with other bacteria. Presterl et al*.*^84^ described negligible bactericidal activity against biofilm-producer *Staphylococcus epidermidis.* The combination also showed killing activity at 24 h by TKA against *Neisseria gonorrhoeae,* including azithromycin resistant strains, with no regrowth until the end of the assay^85^. The latter study is in agreement with our results in *K. pneumoniae*, supporting the potential use of azithromycin/fosfomycin against Gram-negative bacteria (Fig. 4, Figure S4). Interestingly, effective fosfomycin concentrations in our in vitro assays were below fosfomycin peak plasma concentration after intravenous administration in adults (606 mg/L)^86^. To the best of our knowledge, this is the first study analysing the antimicrobial activities of the combination azithromycin/fosfomycin against a panel of MDR *K. pneumoniae* strains*.* Both drugs display good safety profiles, they are recommended for combinatorial therapy to minimize resistance emergence derived from monotherapy, and are administered at a single dose administration (0.5 to 2 g single dose oral or intravenously for azithromycin^73^ and 3 g single dose orally or up to 8 g/8 h intravenously for fosfomycin^87^). Similar to azithromycin, fosfomycin displays immunomodulatory mechanisms^86^, which have been shown beneficial to overcome severe Gram-negative infections. Our results, together with other evidence, suggest that the combination of azithromycin plus fosfomycin could play an important role in clinical settings and merits further pre-clinical and clinical development.

To a lesser extent, in our studies azithromycin also showed interaction with colistin and tigecycline (Fig. 4, Figure S4). The combination with colistin has been reported in some studies including MDR *K. pneumoniae*^82, 88, 89^, where the increase in the Gram-negative outer membrane permeability facilitates azithromycin access to the 50S ribosomal subunit^82, 88^. These findings support the possibility to decrease colistin concentrations below its nephrotoxic threshold (2.42 mg/L)^90^, if administered together with azithromycin. The combination with tigecycline is the first report against *K. pneumoniae*; previous studies described the in vitro and in vivo activity of azithromycin in combination with minocycline (another tetracycline antibiotic) against MDR pathogens including *K. pneumoniae*^91^. However, azithromycin/tigecycline were cytotoxic (Fig. 7), which might raise a concern for potential clinically use. Azithromycin and tigecycline are both bacteriostatic drugs targeting the 50S and 30S ribosomal subunits, respectively, which could explain their synergy by enhancing protein inhibition that leads to disruption of the bacterial gene translation. In fact, the underlying mechanism of action of azithromycin in combination with the three last-line antibiotics tested in this study might follow the same rationale as with zidovudine, i.e., while fosfomycin and colistin enhance intracellular accumulation of both zidovudine and azithromycin due to their cell wall targeting properties, this is not observed with tigecycline.

Azithromycin safety profile is well described, showing uncommon side-effects associated to long-term therapy^92^, and well tolerated when administered to children and pregnant women^93^. It poses advantageous PK/PD properties respect to other macrolides such as no interaction with CYP3A4 cytochrome, an increased tissue penetration and bioavailability due to a higher basic character, and a long half-life (50–70 h)^72, 73^. Peak plasma concentrations of 1.46 mg/L and up to 3.4 mg/L are attained after 1500 mg-oral and 500 mg-intravenous administrations, respectively^72^. In our study, we observed effective sterilizing activities of azithromycin-based combinations at azithromycin concentrations ranging from 2 up to 64 mg/L (Fig. 4, Figure S4), and non-cytotoxic effect below 64 mg/L (Fig. 7). For some strains the azithromycin sterilizing concentrations observed were over those achievable in plasma; however, azithromycin displays a rapid blood-tissue distribution with higher tissue concentrations (e.g. accumulation in macrophages is 5- to 200-fold higher than in plasma^73^). In addition, the long post-antibiotic effect and significant subinhibitory concentration effect demonstrated both in vitro and in vivo against respiratory pathogens^94, 95^ indicate a prolonged antimicrobial activity. As consequence, azithromycin seems an optimal candidate for combination therapy in MDR Gram-negative infections. Standard dosing of the last-line antibiotics used in this study (that included loading doses for colistin and tigecycline)^38^ yielded a rapid bacterial killing effect that could be seconded by the slower but longer lasting action of azithromycin, maintaining bacterial eradication during the course of treatment. Moreover, combinatorial therapy with azithromycin might minimise resistance emergence and toxicity issues (specially with colistin) using longer dosing intervals. The use of macrolides (specially azithromycin) is currently recommended in critically ill patients with pneumonia as empirical treatment in combination with β-lactams or fluroquinolones^96^, supported by previous preclinical assays showing synergy^97–99^. Anticipatory immunotherapy with azithromycin has been also used in critically ill patients with infections other than pneumonia, demonstrating clinical benefit with reduced mortality rates and intensive-care unit (ICU) stay^74^. The early addition of azithromycin to last-line antibiotics for MDR treatment in severe infections (i.e., sepsis, ventilator-associated pneumonia, immunocompromised patients) could not only improve the efficacy of the therapy in combination, but also improve the clinical outcome due to immunomodulatory properties of azithromycin in ICU patients.

In summary, we identified novel synergistic combinations against *K. pneumoniae* adapting to Gram-negative bacteria the mycobacterial sHTSS methodology^14^, using novel validation endpoints in TKA and cytotoxic assays. We demonstrated in vitro that zidovudine and azithromycin are promising repurposing options for the eradication of MDR/XDR *K. pneumoniae.* Based on our in vitro studies, we propose the following priority list of pairwise combinations for further translational studies: azithromycin/fosfomycin = zidovudine/ceftazidime-avibactam > zidovudine/fosfomycin > azithromycin/colistin > fosfomycin/colistin > zidovudine/colistin > meropenem/ertapenem > meropenem/colistin > > zidovudine/tigecycline > fosfomycin/tigecycline > azithromycin/tigecycline. Additional pre-clinical and clinical studies will be needed to fully understand the clinical potential of zidovudine and azithromycin as synergistic partners, especially in patients with severe infections.

### Supplementary Information

Supplementary Tables.

Supplementary Information.

## Data Availability

All data pertaining to this work is within the main manuscript or supplementary information. Primary data are available from the corresponding author upon request.
