# Supplementary material for: In vitro synergy screens of FDA-approved drugs reveal novel zidovudine- and azithromycin-based combinations with last-line antibiotics against Klebsiella pneumoniae
Source: Sci Rep. 2023 Sep 2;13:14429. doi: 10.1038/s41598-023-39647-9 (PMC10475115; doi:10.1038/s41598-023-39647-9)
Supplement: Supplementary file 2 — Supplementary Information. [file 41598_2023_39647_MOESM2_ESM.pdf]

SUPPLEMENTARY FIGURES & TABLES

In vitro synergy screens of FDA-approved drugs reveal novel zidovudine- and azithromycin-based combinations with last-line antibiotics against Klebsiella pneumoniae

Gómara-Lomero, M. *et al.*

SUPPLEMENTARY FIGURES

Figure S1. Representative plate analysis of a semi-High-Throughput Synergy Screen (sHTSS). FDA compounds were pin-spotted onto a soft agar lawn of *K. pneumoniae* in the absence (control plate) or in the presence of subinhibitory concentrations of fosfomycin (16 and 32 mg/L; 1/8xMIC and 1/4xMIC, respectively). Compounds whose zones of inhibition were larger in the presence of fosfomycin than in plates without fosfomycin (examples A, B or C) were selected as hits for further validation of their potential synergistic interaction. High-density plates overlapping inhibition zones for two or more compounds (D, inhibition zones >15 mm) were deconvoluted at a lower compound density or lower concentration (0.1 mM) to allow for clear inhibition zone readings. Ø, inhibition zone diameter value; Red arrows, inhibition zone diameter; r, inhibition zone radius (when diameter cannot be determined); MIC_{FOF}= 128 mg/L.

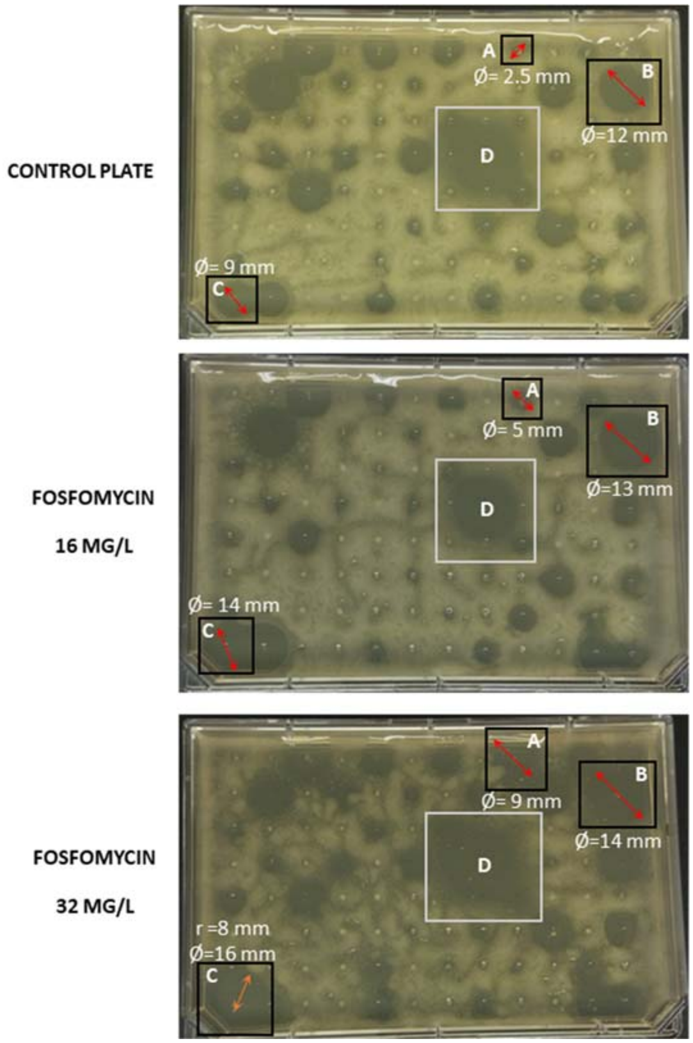

Figure S2. Time-kill curves of pairwise combinations currently used in the therapy of MDR enterobacteria against twelve *K. pneumoniae* strains (a) Meropenem plus ertapenem; **(b)** Meropenem plus colistin; **(c)** Fosfomycin plus colistin; **(d)** Fosfomycin plus tigecycline

a) Meropenem plus ertapenem

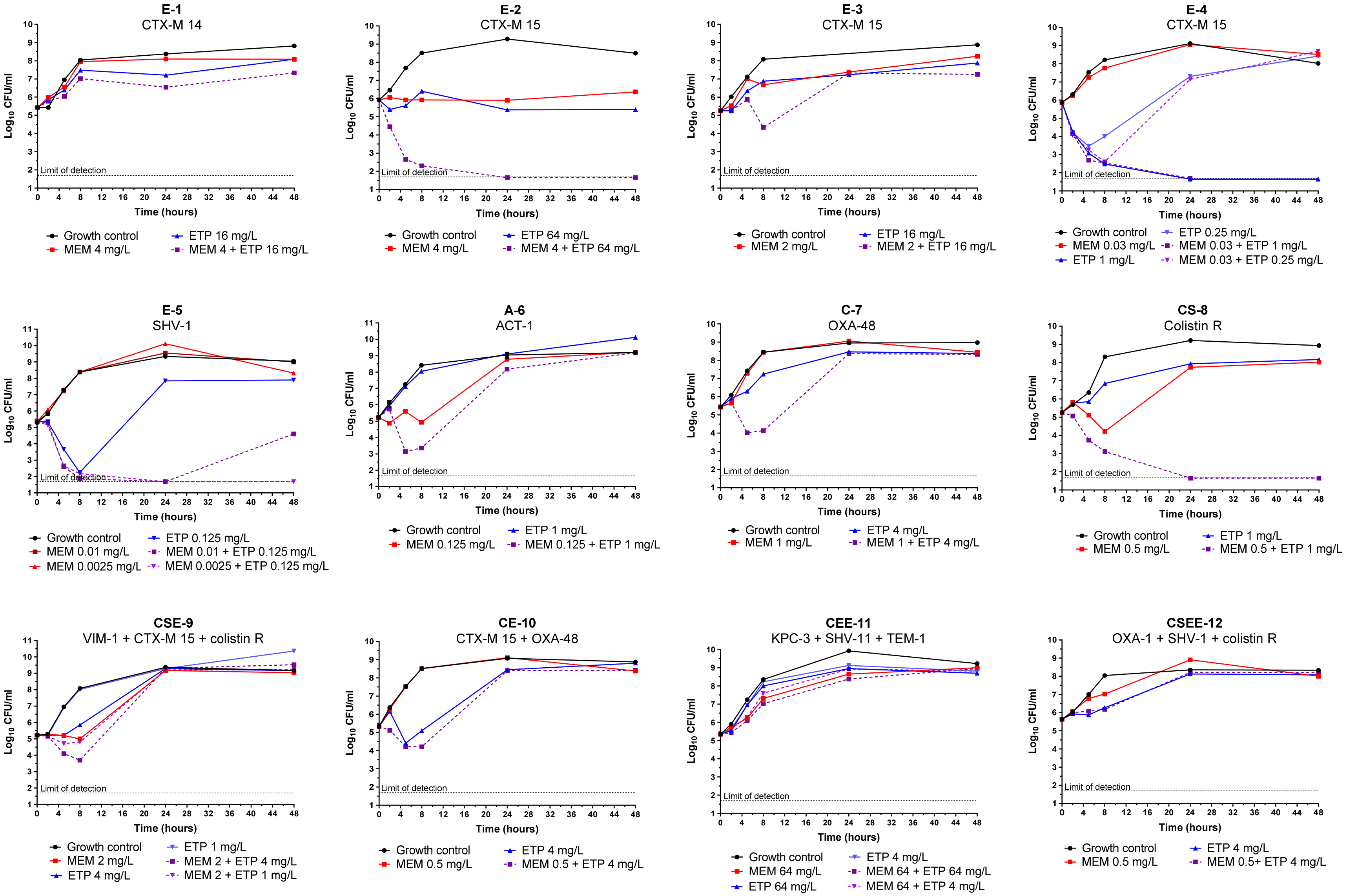

b) Meropenem plus colistin

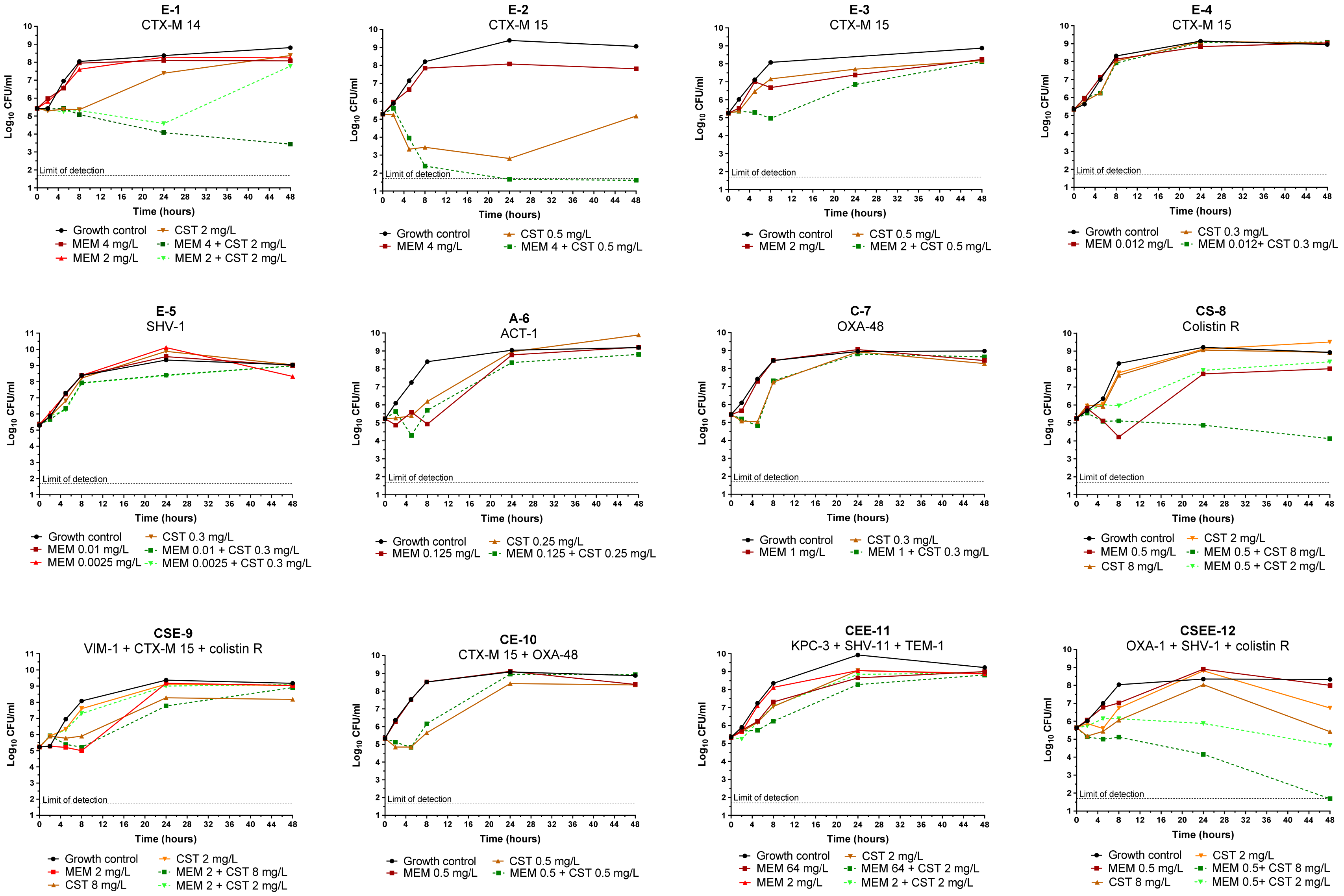

c) Fosfomycin plus colistin

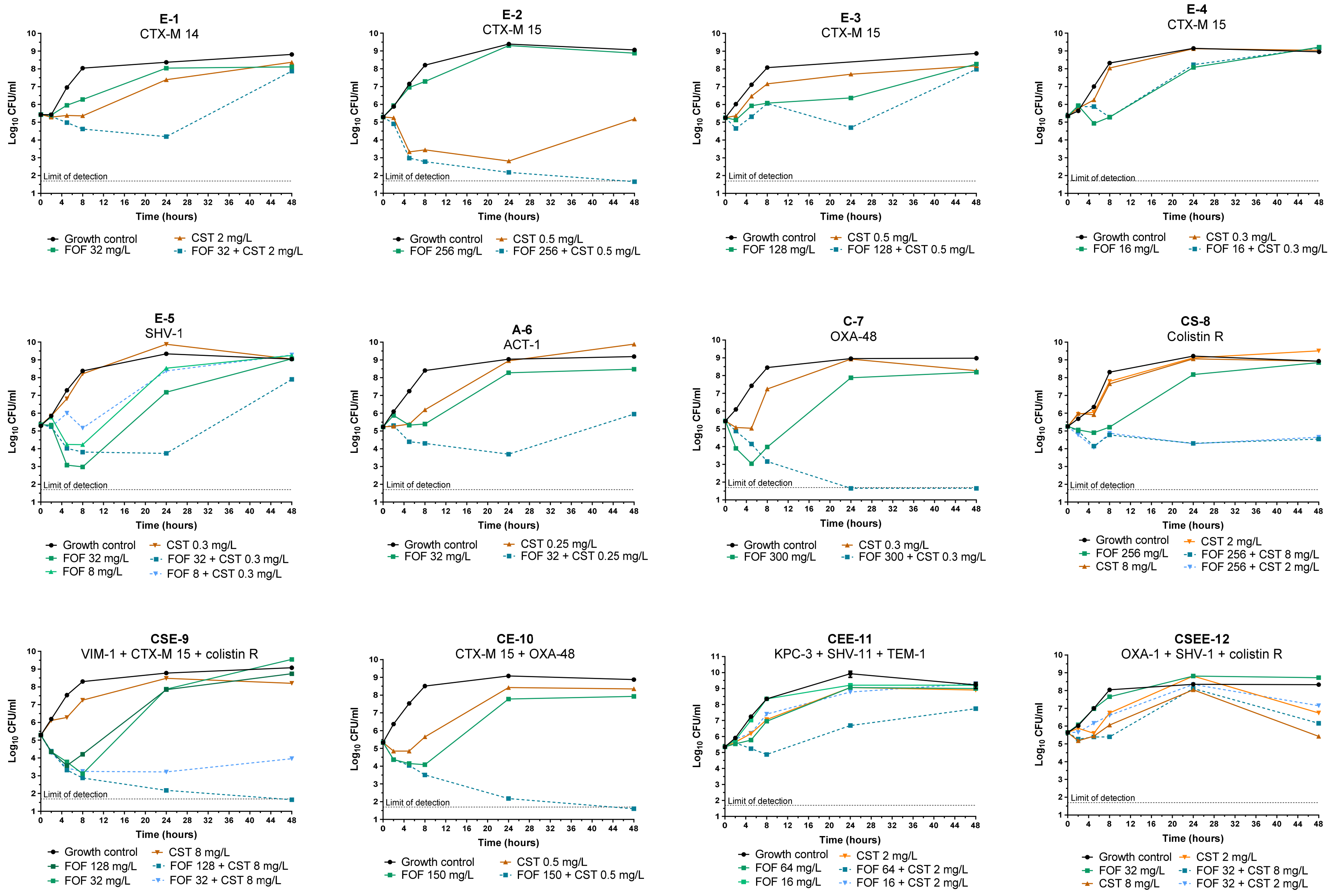

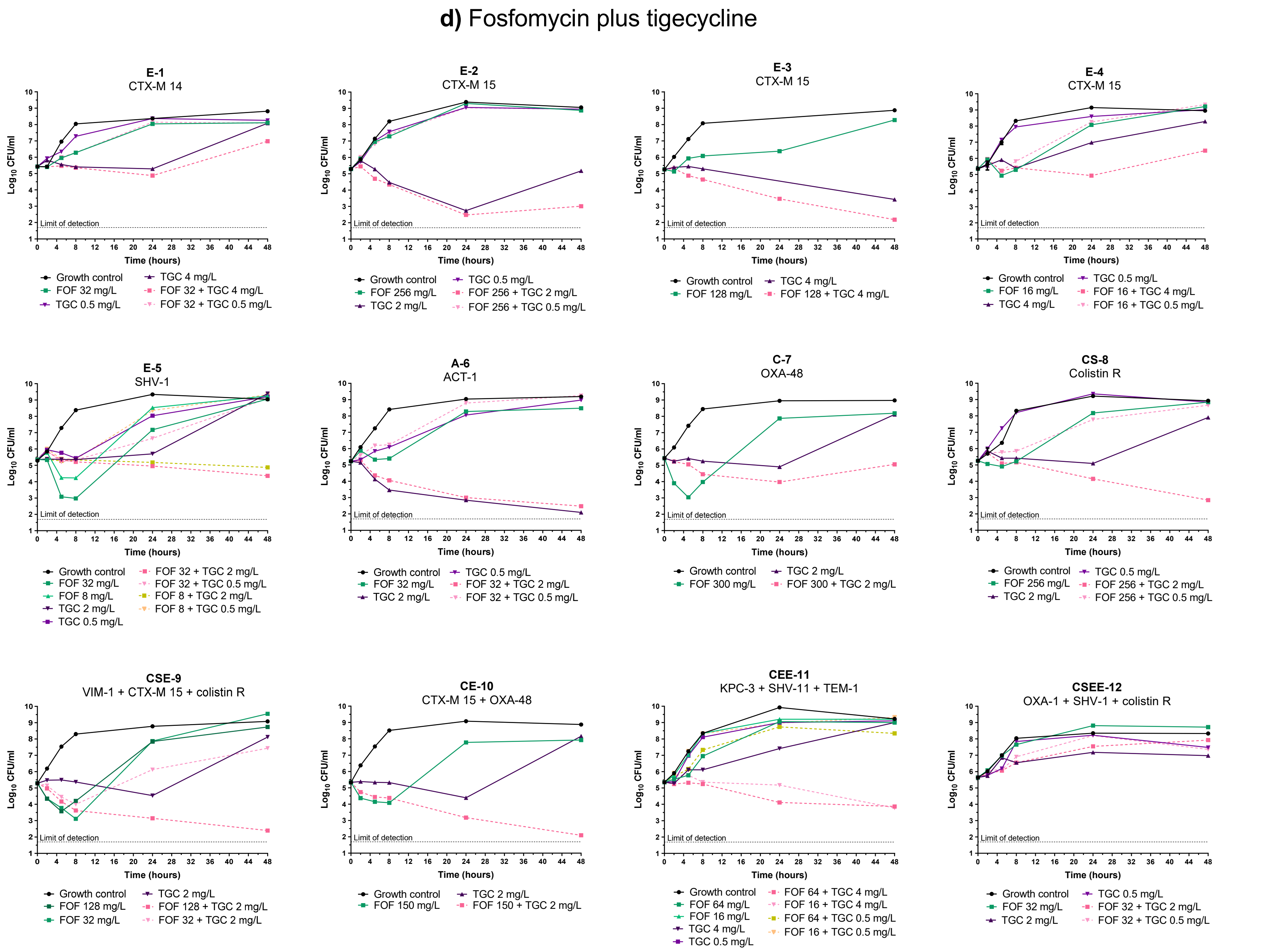
d) Fosfomycin plus tigecycline
E-1
CTX-M 14
E-2
CTX-M 15
E-3
CTX-M 15
E-4
CTX-M 15
E-5
SHV-1
A-6
ACT-1
C-7
OXA-48
CS-8
Colistin R
CSE-9
VIM-1 + CTX-M 15 + colistin R
CE-10
CTX-M 15 + OXA-48
CEE-11
KPC-3 + SHV-11 + TEM-1
CSEE-12
OXA-1 + SHV-1 + colistin R
Log10 CFU/ml
Time (hours)
Limit of detection
Growth control
FOF 32 mg/L
TGC 0.5 mg/L
TGC 4 mg/L
FOF 32 + TGC 4 mg/L
FOF 32 + TGC 0.5 mg/L
FOF 256 mg/L
TGC 2 mg/L
FOF 256 + TGC 2 mg/L
FOF 256 + TGC 0.5 mg/L
FOF 128 mg/L
FOF 128 + TGC 4 mg/L
FOF 16 mg/L
FOF 16 + TGC 4 mg/L
FOF 16 + TGC 0.5 mg/L
FOF 8 mg/L
FOF 32 + TGC 2 mg/L
FOF 8 + TGC 2 mg/L
FOF 8 + TGC 0.5 mg/L
FOF 300 mg/L
FOF 300 + TGC 2 mg/L
FOF 128 + TGC 2 mg/L
FOF 150 mg/L
FOF 150 + TGC 2 mg/L
FOF 64 mg/L
FOF 64 + TGC 4 mg/L
FOF 16 + TGC 4 mg/L
FOF 64 + TGC 0.5 mg/L
FOF 16 + TGC 0.5 mg/L

Figure S3. Time-kill assays of zidovudine combined with last-line antibiotics against twelve *K. pneumoniae* strains. (a) Zidovudine plus ceftazidime-avibactam; **(b)** Zidovudine plus fosfomycin; **(c)** Zidovudine plus colistin; **(d)** Zidovudine plus tigecycline

a) Zidovudine plus ceftazidime-avibactam

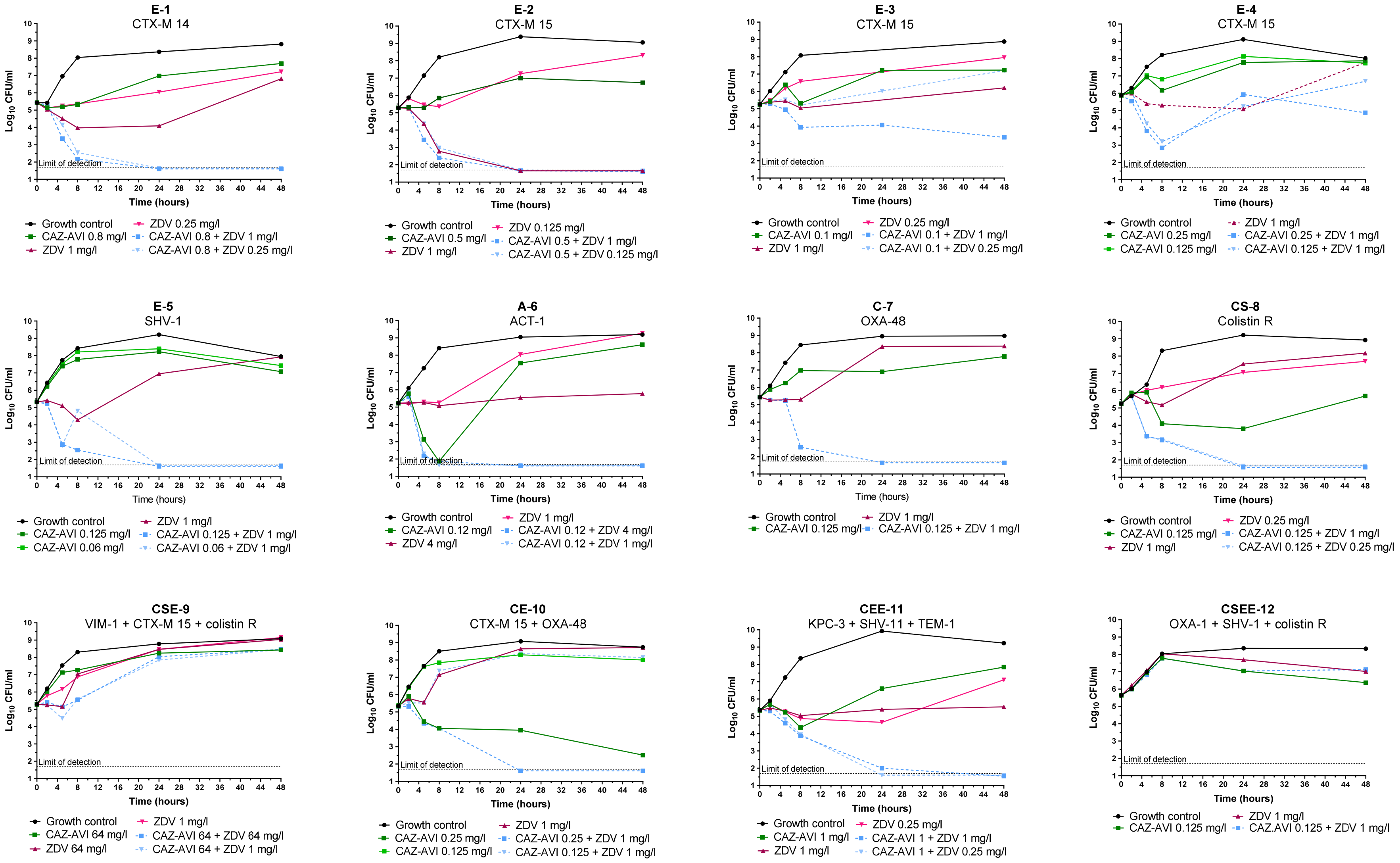

b) Zidovudine plus fosfomycin

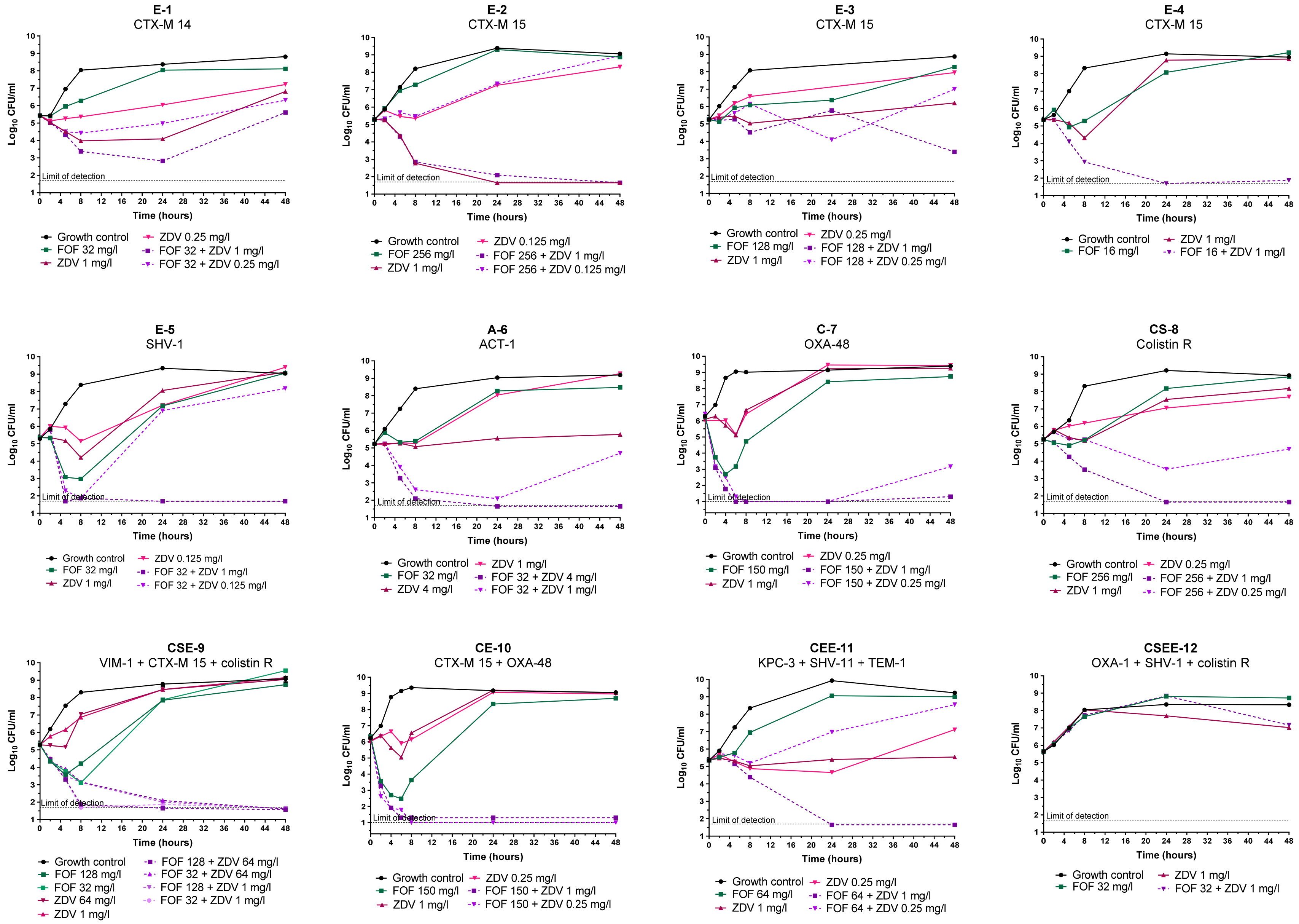

c) Zidovudine plus colistin

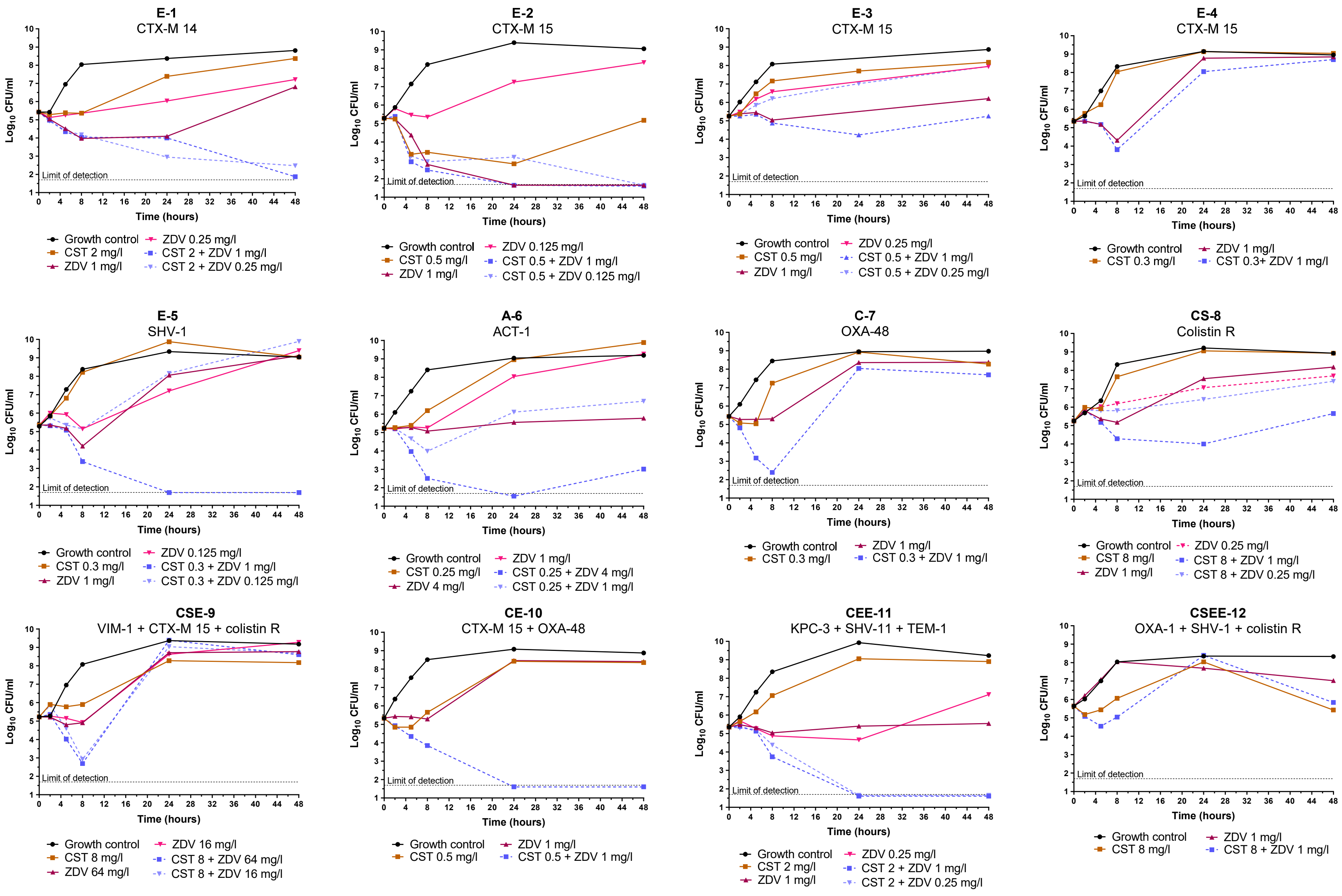

d) Zidovudine plus tigecycline

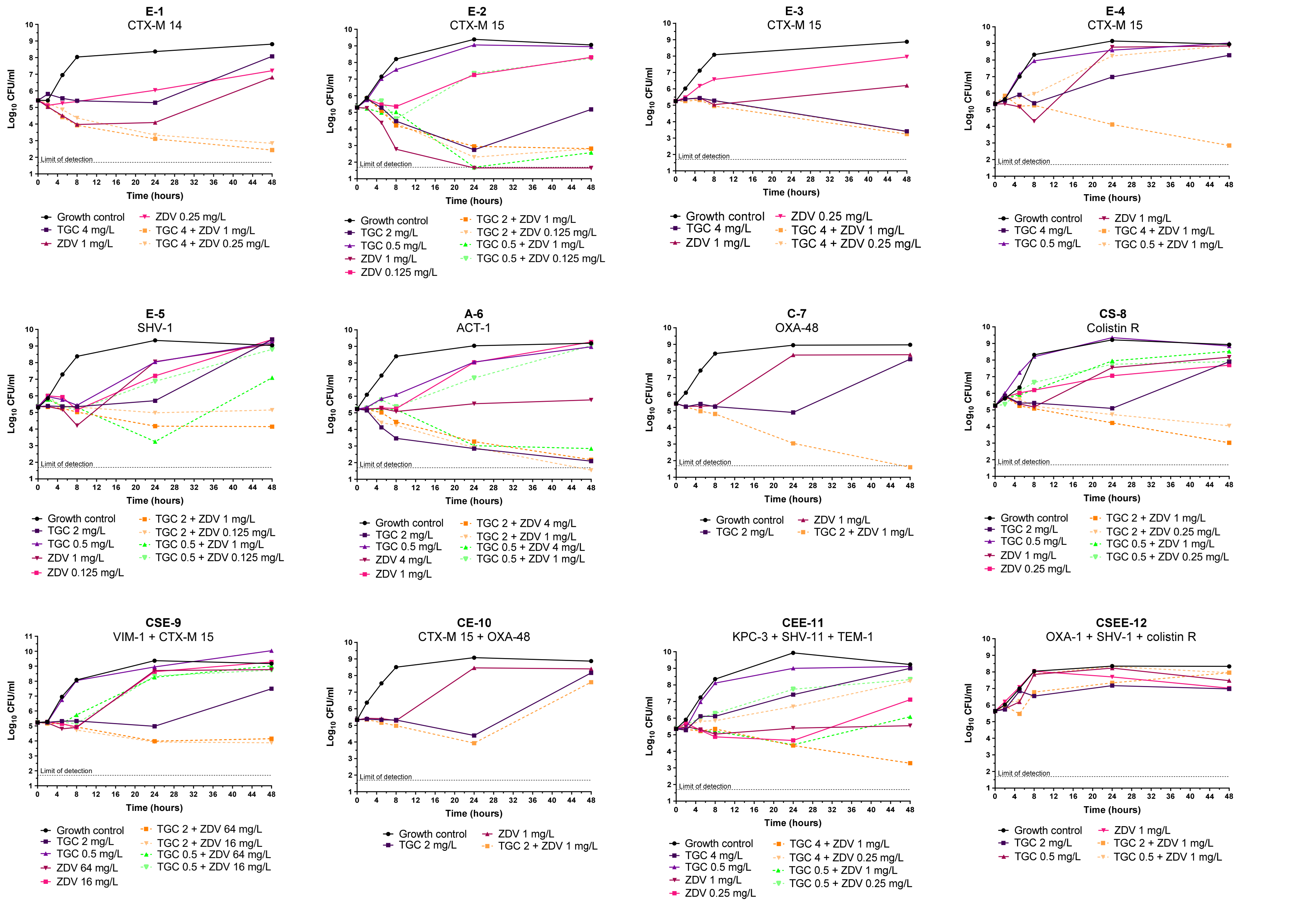

Figure S4. Time-kill assays of azithromycin combined with last-line antibiotics against twelve *K. pneumoniae* strains. (a) Azithromycin plus fosfomycin; **(b)** Azithromycin plus colistin; **(c)** Azithromycin plus tigecycline.

a) Azithromycin plus fosfomycin

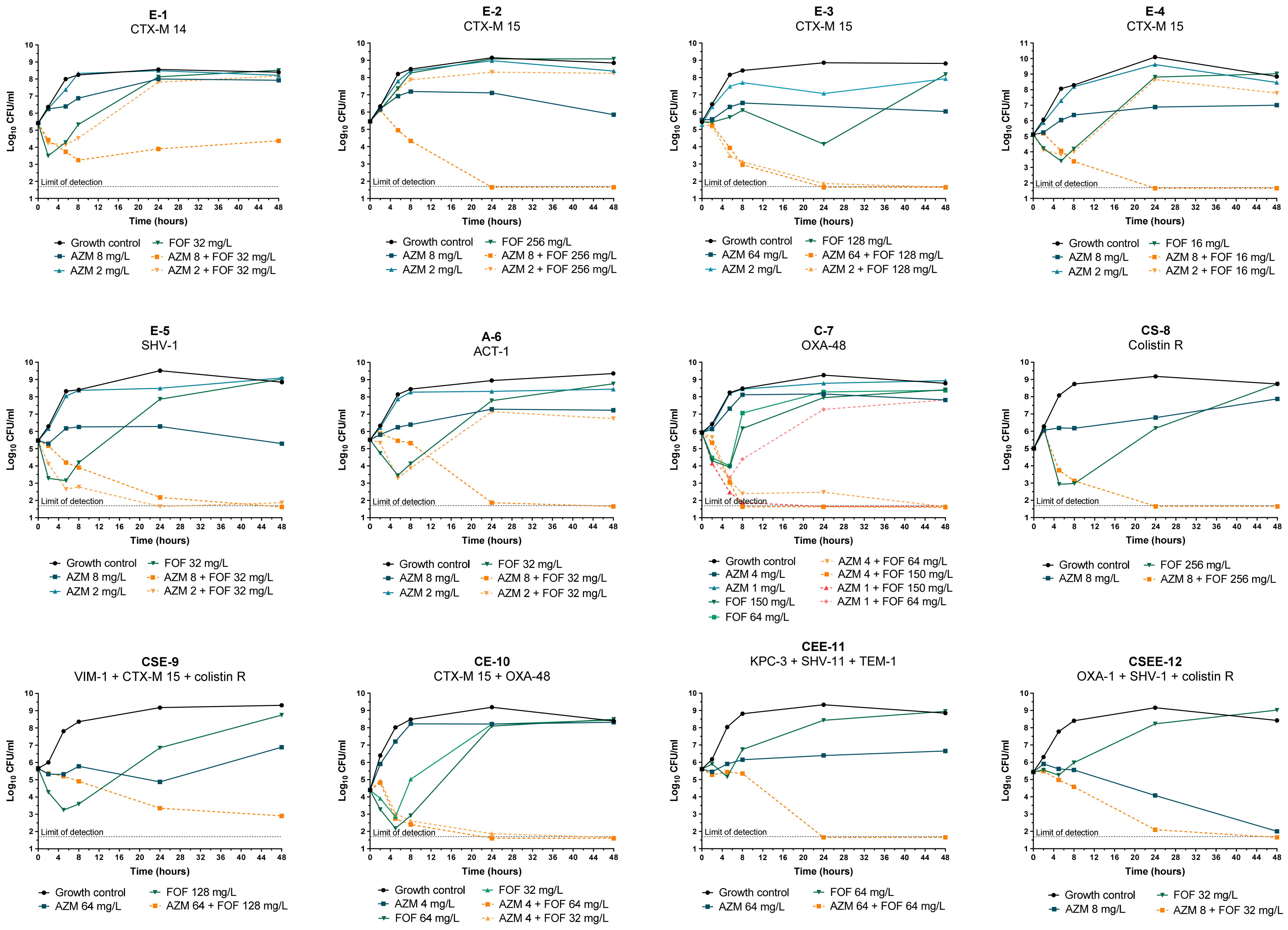

b) Azithromycin plus colistin

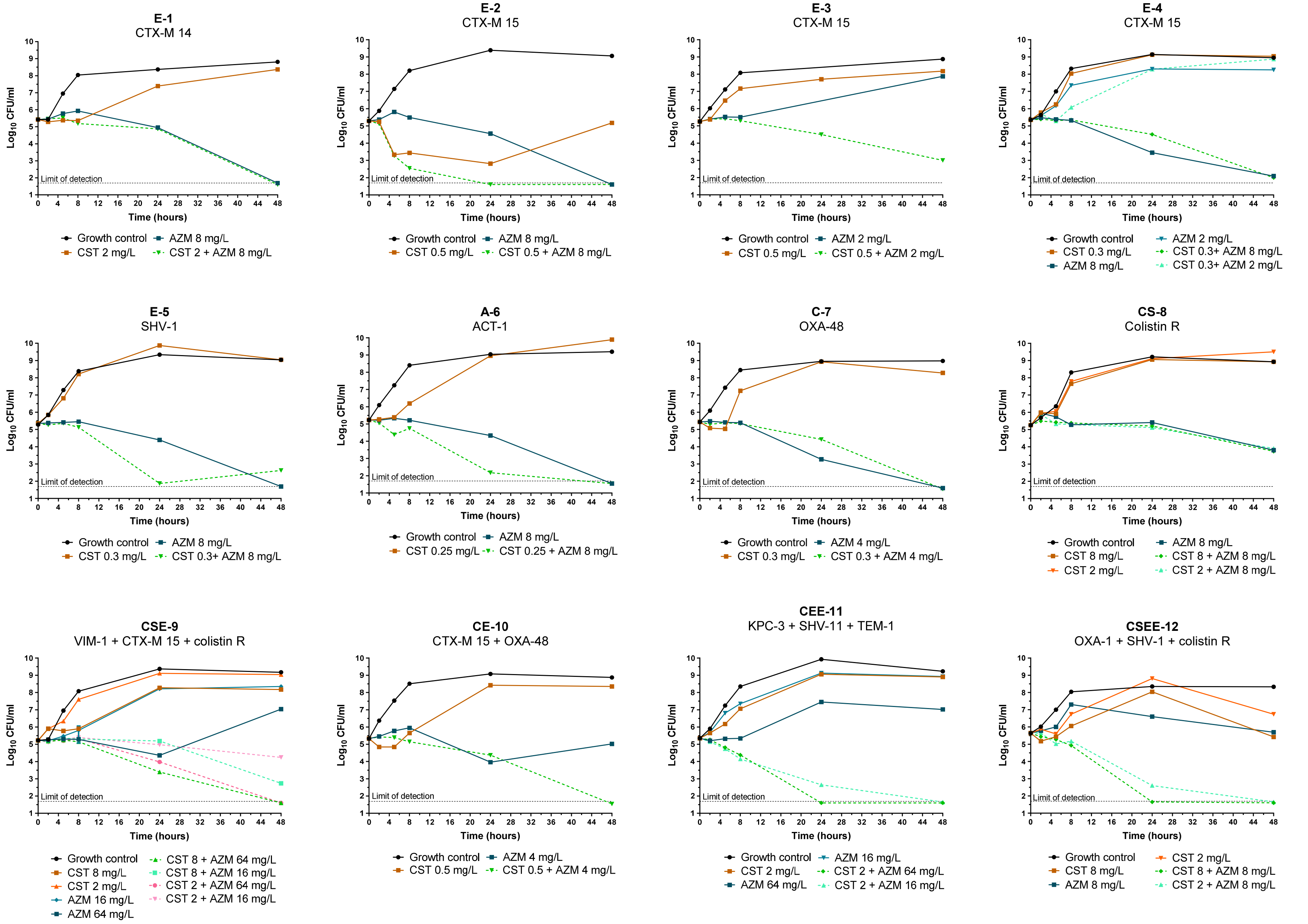

c) Azithromycin plus tigecycline

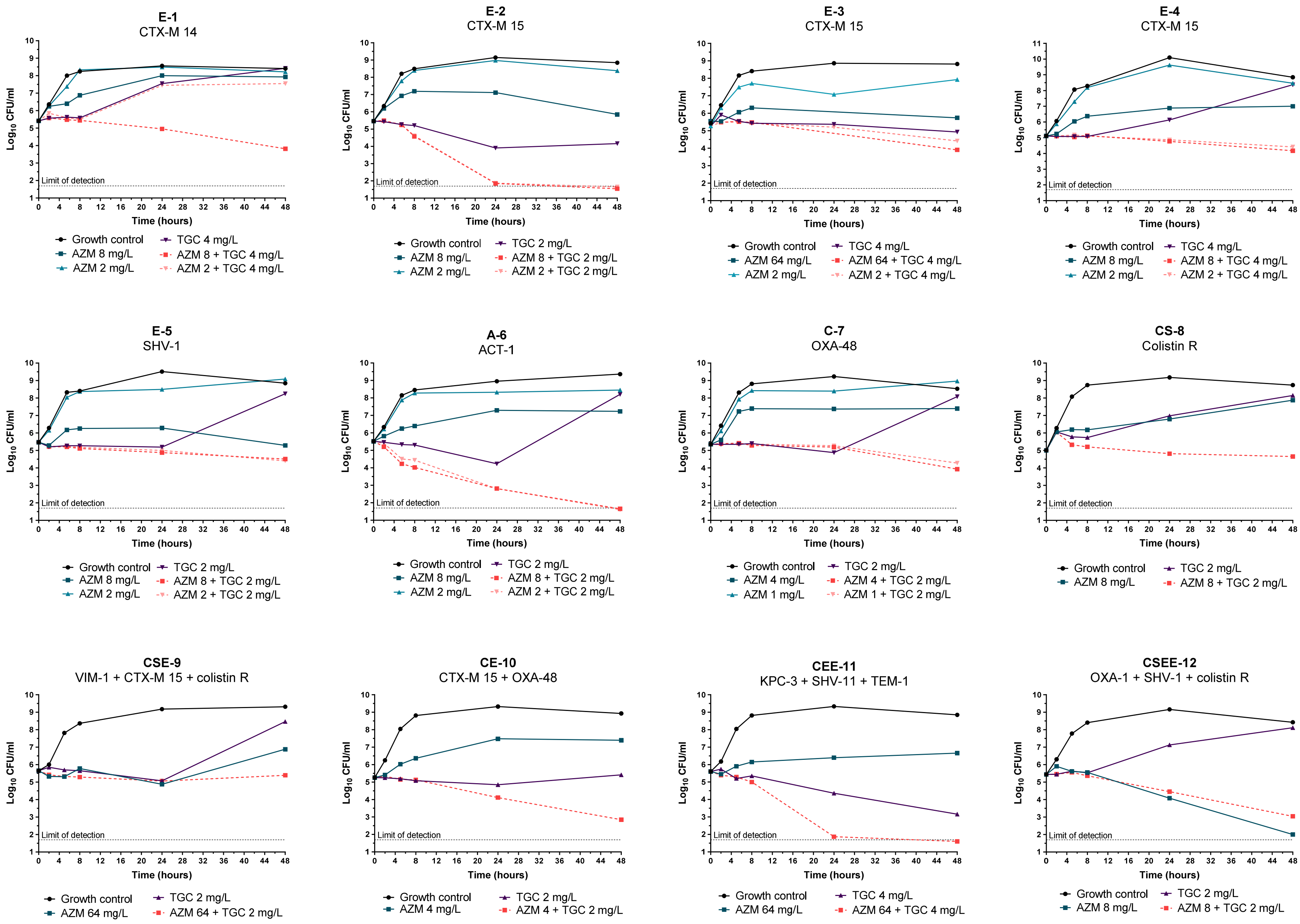

Figure S5. Time-kill curves of zidovudine, colistin and fosfomycin alone, pairwise and triple combination against eight *K. pneumoniae* isolates.

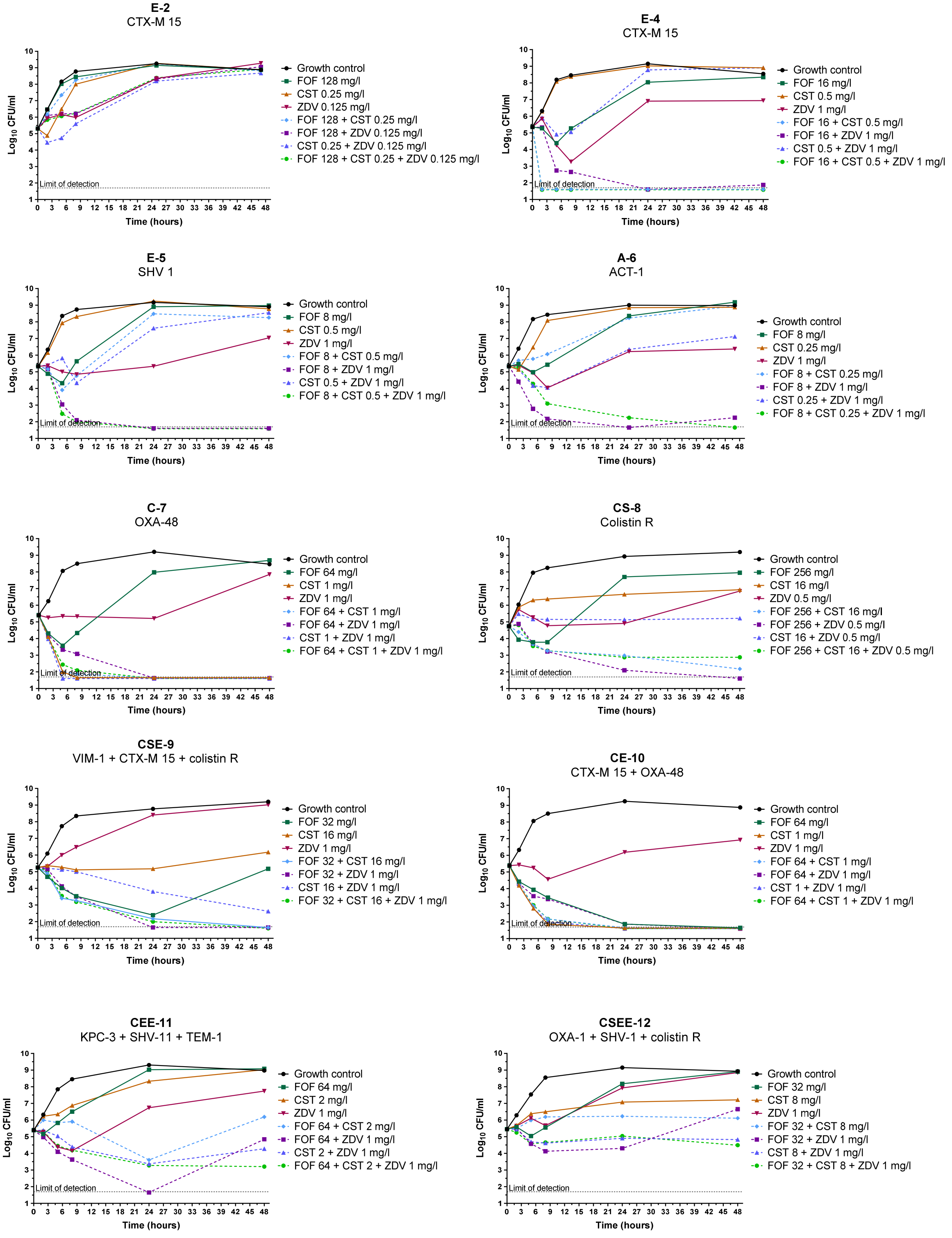

Figure S6. Secondary validation CBA and TKA of tigecycline in combination with aztreonam against *K. pneumoniae* ATCC 13883. (a) CBA were performed considering the MIC and MBC values of each compound alone and in combination. The FICI and FBCI were calculated from the most optimal combinatorial concentration (lowest value in the combination, red squares). Based on these assays, the combination was classified as “no interaction” (FICI/FBCI values between 0.5-4). **(b)** TKA provided longitudinal information showing a reduction of 1.01 log_{10} in CFU/mL of the combination with respect to tigecycline alone at 24 hours; thus, at this time point, the interaction was not classified as synergistic (<2 log_{10} reduction in CFU/mL with respect to the most active drug). However, after 48 hours the combination could be classified as synergistic with a reduction of 6.65 log_{10} CFU/mL with respect to tigecycline alone, and prevention of bacterial re-growth (proxy for sterilizing activity). MIC_{ATM} = 0.125-0.25 mg/L; MIC_{TGC} = 0.8 mg/L; MBC_{ATM} = 0.25 mg/L; MBC_{TGC} = 1.6 mg/L. CBA, checkerboard assay; TKA, time-kill assay; TGC, tigecycline; ATM, aztreonam.

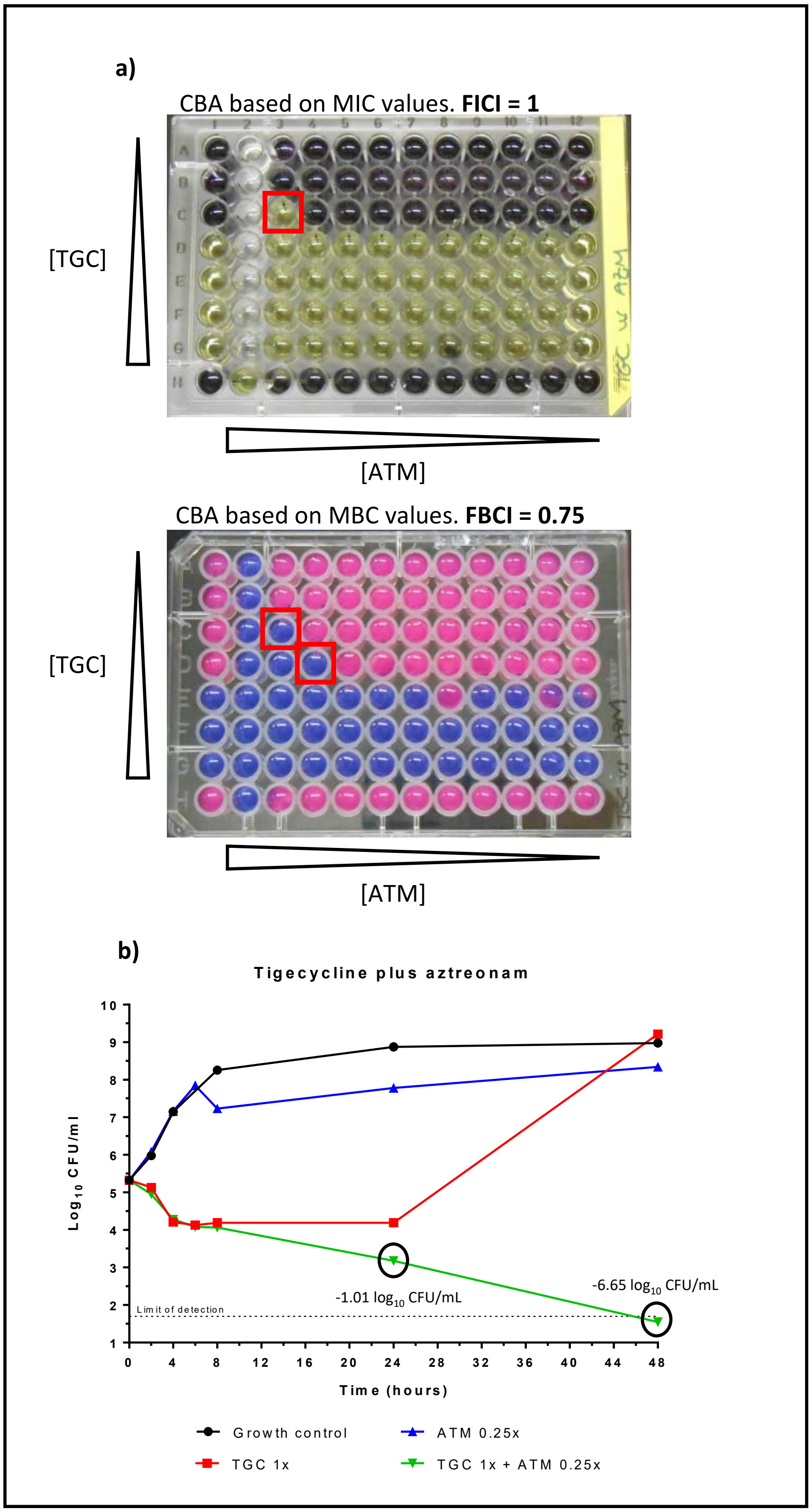
a)
CBA based on MIC values. FICI = 1
[TGC]
[ATM]
CBA based on MBC values. FBCI = 0.75
[TGC]
[ATM]
b)
Tigecycline plus aztreonam
Log10 CFU/ml
Time (hours)
Limit of detection
-1.01 log10 CFU/mL
-6.65 log10 CFU/mL
Growth control
ATM 0.25x
TGC 1x
TGC 1x + ATM 0.25x

SUPPLEMENTARY TABLES

Table S1. Strain characterization and antimicrobial susceptibility of the twelve MDR/XDR *K. pneumoniae* strains used in this study.

				MIC (mg/L)[b]																					
Isolate	Resistance mechanism	Specimen source	MDR/XDR classification[a]	AMK	GEN	TOB	AMP/AMX	AMC	TZP	FOX	CXM	CTX	CAZ	FEP	ATM	IPM	ETP	MEM	CIP	LVX	FOF	CST	TGC	SXT
E-1	CTX-M 14	Rectal swab	XDR	≤8	>8	≤2	>16	>16/8	>64	>16	>16	>32	>16	>16	>16	8	>1	8	>2	4	≤32	≤2	>2	>4/76
E-2	CTX-M 15	Blood	MDR	≤8	≤2	>8	>16	>16/8	>64	>16	>16	>32	>16	>16	>16	≤1	>1	2	>2	>4	>64	≤2	≤1	≤2/38
E-3	CTX-M 15	Abscess	MDR	≤8	>8	>8	>16	>16/8	64	>16	>16	>32	>16	>16	>16	≤1	>1	2	>2	2	≤32	≤2	≤1	>4/76
E-4	CTX-M 15	Blood	MDR	16	>4	>4	>16	>32	16	>16	>8	>32	32	>8	>4	≤1	≤0.12	≤0.12	>1	>1	≤16	≤2	>2	>4/76
E-5	SHV-1 + porin loss	Blood	MDR	≤8	≤2	≤2	>16	>16/8	>64	16	≤4	≤1	≤1	4	≤1	≤1	≤0.5	≤1	≤0.5	≤1	≤32	≤2	≤1	≤2/38
A-6	AmpC ACT-1	SEIMC CCS07	MDR	>32	>8	>8	>16	>16/8	64	>16	>16	32	>16	≤1	>16	≤1	>1	≤1	2	≤1	≤32	≤2	≤1	>4/76
C-7	OXA-48	Blood	MDR	≤8	≤2	≤2	>16	>16/8	>64	≤8	8	≤1	≤1	≤1	≤1	4	>1	4	≤0.5	≤1	64	≤2	≤1	≤2/38
CS-8	Colistin R	Urine	MDR	≤8	≤2	≤2	>16	>16/8	>64	>16	16	≤1	≤1	4	≤1	≤1	≤0.5	≤1	≤0.5	≤1	>64	>4	2	>4/76
CE-9	VIM-1 + CTX-M 15 + colistin R	SEIMC CCS04	XDR	16	>8	>8	>16	>16/8	>64	>16	>16	>32	>16	>16	>16	2	>1	8	>2	>4	≤32	>4	2	>4/76
CE-10	CTX-M 15 + OXA-48	Blood	MDR	≤8	>4	>4	>8	>32	>16	≤8	>8	>32	32	>8	>4	8	>1	1	>1	>1	32	≤2	≤1	>4/76
CEE-11	KPC-3 + SHV-11 + TEM-1	SEIMC CCS05	XDR	32	4	>8	>16	>16/8	>64	>16	>16	>32	>16	>16	>16	>8	>1	>8	>2	>4	64	>4	>2	>4/76
CSEE-12	OXA-1 + SHV-1 + colistin R	EARS QC	MDR	>32	>8	>8	>16	>16/8	>64	≤8	>16	>32	≤1	>16	≤1	≤1	>1	≤1	>2	>4	≤32	>4	2	>4/76

[a]MDR/XDR categorization according to Magiorakos *et al.* (15): MDR: non-susceptible to ≥1 agent in ≥3 antimicrobial categories; XDR: non-susceptible to ≥1 agent in all but ≤2 categories

[b]MIC determined by automated broth microdilution (Microscan Walkaway®, Beckman Coulter, Spain) and clinical interpretation according to the corresponding EUCAST guidelines on the isolation date. Values in green were interpreted as "Susceptible", in dark yellow as "Susceptible, increased exposure" (EUCAST 2019), in light yellow as "Intermediate" and in red as "Resistant"

AMK, Amikacin; GEN, Gentamicin; TOB, Tobramycin; AMP/AMX, Ampicillin/amoxicillin; AMC, Amoxicillin-clavulanate; TZP, Piperacillin-tazobactam; FOX, Cefoxitin; CXM, Cefuroxime; CTX, Cefotaxime; CAZ, Ceftazidime; FEP, Cefepime; ATM, Aztreonam; IPM, Imipenem; ETP, Ertapenem; MEM, Meropenem; CIP, Ciprofloxacin; LVX, Levofloxacin; FOF, Fosfomycin; CST, colistin; TGC, Tigecycline; SXT, Trimethoprim/sulfamethoxazole

Table S2. MIC values of colistin, fosfomycin and tigecycline determined by three different methods. Concentrations are expressed in mg/L.

	**MIC (mg/L) against *K. pneumoniae ATCC 13883***		
	**Broth microdilution**	**Agar dilution**	**sHTSS**
Tigecycline	0.5	0.5	0.5
Colistin	1	1	0.03
Fosfomycin	128	128	128

Table S3. FDA compounds identified in the sHTSS with tigecycline, colistin and fosfomycin and validation data against *K. pneumoniae* ATCC 13883

See supplementary excel file

Table S4. Drug susceptibility of *K. pneumoniae* ATCC 13883 to the FDA compounds showing synergy with tigecycline, colistin or fosfomycin. [a]Determined by MTT assay. [b]Determined by resazurin assay.

Compound	MIC values (mg/L)[a]	MBC values (mg/L)[b]
Amikacin	1	2
Azithromycin	2	2-4
Aztreonam	0.125	0.25
Balofloxacin	0.25	0.25
Bleomycin	0.25	0.25
Cefdinir	0.5	0.5
Cefmenoxime	0.25	0.25-0.5
Cefoperazone	4	4
Ceftazidime	0.5	0.5
Ceftiofur	1	1
Ceftriaxone	0.125	0.125
Cephradine	32	32
Colistin	1-2	1-2
Danofloxacin	0.06	0.12
Difloxacin	0.12-0.25	0.25-0.5
Doripenem	0.03	0.06
Doxycycline	4	>8
Enrofloxacin	0.015-0.03	0.03-0.06
Flumequine	1-2	1-2
Fosfomycin	≥128	≥128
Furazolidone	1	1
Ibandronate	>32	>32
Ivermectin	>64	>64
Levofloxacin	0.03	0.03
Lomefloxacin	0.12-0.25	0.12
Marbofloxacin	0.015-0.03	0.015-0.06
Methacycline	1	32
Moxalactam	0.5	0.5
Moxifloxacin	0.25-0.5	0.25-0.5
Nadifloxacin	1	1
Netilmicin	1	1
Norfloxacin	2	2
Ofloxacin	0.125	0.125
Pefloxacin	0.125	0.125
Penfluridol	>32	>32
Pralidoxime	>32	>32
Rifaximin	16	32
Sisomicin	0.125	0.25
Sparfloxacin	0.03	0.03
Streptomycin	2	2
Terbutaline	>32	>32
Tigecycline	0.5	1?
Tobramicin	0.125	0.125
Triclosan	0.25-0.5	0.25
Trimetoprim	1	8-16
Zidovudine	0.06	0.5-0.12

Table S5. Screening and validation of synergistic combinations against *K. pneumoniae* ATCC 13883.

	Primary Compound	Tigecycline			Colistin			Fosfomycin			Grand total (three PCs)		
		S	A	Total	S	A	Total	S	A	Total	S	A	Total
	sHTSS[a]	37*	6	43	31*	10	41	41*	12	53	**109**	**28**	137
Secondary validation	CBA assayed	14	2	16	11	1	12	25	0	25	50	3	53
	CBA validated[b]	0	0	0	6 + 1#	-	7	1	0	1	8	0	8
	TKA assayed	10	3	13	8	3	11	17	0	17	35	6	41
	TKA validated[c]	7	0	7	5 + 3#	-	8	12	0	12	27	0	27
Novelty	Already published combinations	3	-	-	3	-	-	2	-	-	8	-	-
	Novel combinations (non-antibiotics)	2	-	-	2	-	-	3	-	-	7	-	-
	Novel combinations (other)	2	-	-	3	-	-	7	-	-	12	-	-

[a]Interaction classification was based on the increment of the inhibition zones at the two PCs sub-inhibitory concentrations tested compared to those of the no PC plates as described in Material and Methods. Raw data are displayed, including compounds with different chemical forms (i.e., amikacin hydrate / amikacin disulfate)

[b]FICI or FBCI ≤0.5 indicates synergy

[c]Synergy was defined as a ≥2 log_{10} reduction in CFU/mL in the combination compared to the most active compound alone

*Synergistic interactions included those classified as synergy (Y) and likely synergy (Y/N)

#Interaction with bleomycin validated as synergy by CBA or TKA although sHTSS initially identified antagonistic interaction

S: synergy; A; antagonism; sHTSS, semi-high throughput synergy screen; CBA, checkerboard assays; TKA, time-kill assays

Table S6. Time-kill assays drug interaction data against *K. pneumoniae* ATCC 13883. This table includes the raw data supporting **Figure 2**. Data display the number of residual viable colonies (Δ$\log_{10}$ CFU/mL) between the combination and the most active agent alone and between the initial and final inoculum at the different time points (after 8, 24 and 48 hours of incubation). A negative sign denotes a reduction in the viable counts. Values in bold: synergistic (≥2 $\log_{10}$ CFU/mL reduction) and bactericidal effects (≥3 $\log_{10}$ CFU/mL reduction). Initial inoculum was $5x10^5$ CFU/mL. The experimental limit of detection was 50 CFU/mL.

		Δlog10 CFU/mL between the combination and the most active agent			Δlog10 CFU/mL between initial and final inoculum		
**Combinations**	**Concentrations (mg/L)**	**8 h**	**24 h**	**48 h**	**8 h**	**24 h**	**48 h**
**TGC / AZM**	0.5 / 2	-0.99	**-2.50**	**-6.01**	-2.12	**-3.63**	**-3.63**
**TGC / ATM**	0.5 / 0.025	-0.13	-1.01	**-6.65**	-1.26	-2.15	**-3.63**
**TGC / RAD**	0.5 / 8	-0.08	-1.24	**-5.93**	-1.21	-2.37	-2.34
**TGC / ENR**	0.5 / 0.003	0.36	-0.69	-0.74	-1.35	0.93	2.26
**TGC / FZD**	0.5 / 0.25	2.48	-0.70	0.03	-0.82	2.43	3.40
**TGC / IBN**	0.5 / 8	1.13	0.71	**-4.04**	0	-0.42	-1.08
**TGC / IVM**	0.5 / 64	-0.07	-0.44	0	0.48	2.68	3.37
**TGC / LVX**	0.5 / 0.03	-0.17	**-4.00**	**-2.95**	-1.46	-2.57	0.18
**TGC / MET**	0.5 / 1	-0.04	-0.32	-1.74	-1.18	-1.45	2.15
**TGC / PFD**	0.5 / 8	1.04	0.74	**-2.00**	-0.09	-0.39	0.49
**TGC / STP**	0.5 / 0.5	-0.91	-0.47	-0.73	-0.36	2.65	2.40
**TGC / TOB**	0.5 / 0.125	-0.09	-1.19	**-7.23**	-1.23	-2.32	**-3.63**
**TGC / TCS**	0.5 / 0.125	-1.94	-0.43	-0.03	-1.40	2.70	3.34
**CST / AMK**	0.25 / 0.125	-1.98	-0.03	-0.12	-0.95	3.92	3.86
**CST / AZM**	0.25 / 2	**-4.29**	**-6.29**	**-5.24**	**-3.26**	**-3.26**	**-3.26**
**CST / ATM**	0.25 / 0.025	0.02	-0.04	0.12	1.05	3.07	3.65
**CST / BLE**	0.25 / 0.06	**-3.58**	**-7.21**	**-7.26**	-2.56	**-3.26**	**-3.26**
**CST / CRO**	0.25 / 0.03	0.14	0.10	-0.10	1.16	2.89	2.95
**CST / ENR**	0.25 / 0.003	**-3.98**	-1.45	-0.38	-2.95	1.44	3.21
**CST / FZD**	0.25 / 0.25	-0.49	**-7.09**	**-7.24**	**-3.79**	**-3.79**	**-3.79**
**CST / IVM**	0.25 / 64	**-2.91**	-0.64	0.07	-2.78	2.70	3.52
**CST / LVX**	0.25 / 0.03	**-2.50**	**-5.21**	**-6.92**	**-3.79**	**-3.79**	**-3.79**
**CST / TOB**	0.25 / 0.125	**-2.65**	-0.72	-0.56	-2.52	2.62	2.43
**CST / TCS**	0.25 / 0.125	**-3.68**	**-7.21**	**-7.39**	-2.65	**-3.26**	**-3.26**
**FOF / AMK**	128 / 0.125	-0.36	-0.84	0.20	-2.10	2.16	3.34
	32 / 0.125	-0.03	-1.05	1.05	0.23	2.23	4.00
**FOF / AZM**	128 / 2	-1.01	**-6.18**	**-4.37**	-2.74	**-4.01**	**-4.01**
	32 / 2	-1.49	**-6.18**	**-4.37**	-1.22	**-4.01**	**-4.01**
**FOF / BLE**	128 / 0.06	-1.79	**-7.01**	**-7.15**	**-3.52**	**-4.01**	**-4.01**
	32 / 0.06	**-3.57**	**-5.24**	-1.18	**-3.30**	-1.96	1.78
**FOF / CDR**	128 / 0.125	-1.82	**-5.31**	**-6.75**	-2.39	**-3.44**	-2.95
	32 / 0.125	-0.87	-0.22	-0.23	-1.20	2.52	3.57
**FOF / CRO**	128 / 0.03	-0.54	**-2.12**	**-6.24**	-1.11	-0.26	**-3.44**
	32 / 0.03	**-2.61**	0.37	0.63	-0.07	2.71	3.43
**FOF / DOR**	128 / 0.03	0	**-5.74**	**-7.21**	-1.14	-2.95	**-3.26**
	32 / 0.03	-0.71	**-5.34**	**-5.81**	-0.79	-2.56	-1.86
**FOF / DOX**	128 / 4	1.03	-1.71	**-3.85**	-0.11	-2.18	-0.48
	32 / 4	0.05	-0.94	-0.40	0	-1.41	2.98
	128 / 1	0.27	**-2.72**	-0.43	-0.88	0.07	3.74
	32 / 1	-0.12	-0.10	0.03	0.95	3.02	4.21

Combinations	Concentrations (mg/L)	Δlog_{10} CFU/mL between the combination and the most active agent			Δlog_{10} CFU/mL between initial and final inoculum		
		8 h	24 h	48 h	8 h	24 h	48 h
**FOF / ENR**	128 / 0.003	-1.61	-1.07	0.11	-2.18	0.80	3.93
	32 / 0.003	-1.61	-0.20	0.15	0.93	3.32	4.06
**FOF / FZD**	128 / 0.25	-1.56	**-4.52**	-1.11	-2.13	-2.65	2.71
	32 / 0.25	0.30	0	-0.40	1.02	3.17	3.57
**FOF / LMF**	128 / 0.025	**-2.27**	**-6.07**	**-7.15**	**-4.01**	**-3.22**	**-4.01**
	32 / 0.025	-0.79	-1.57	0.28	-0.52	1.28	3.23
**FOF / MOX**	128 / 0.125	-0.27	**-4.62**	**-6.62**	-1.41	**-3.26**	**-3.26**
	32 / 0.125	0.19	1.17	0.8	-0.86	2.52	4.16
**FOF / PRA**	128 / 8	-0.23	-0.10	0.30	-0.80	1.77	4.12
	32 / 8	-0.55	**-2.46**	0.43	1.98	1.89	4.41
**FOF / RFX**	128 / 4	0.12	**-4.90**	-1.80	-1.02	-2.11	2.26
	32 / 4	-0.24	-0.12	-0.05	0.82	3.00	4.12
**FOF / BUT**	128 / 8	0.39	1.00	0.24	-0.18	2.87	4.06
	32 / 8	0.21	-0.15	0.02	2.74	3.85	4.00
**FOF / TCS**	128 / 0.125	0.64	1.18	-0.30	0.07	3.05	3.52
	32 / 0.125	-0.51	-0.30	0.49	1.89	3.47	4.47
**FOF / TMP**	128 / 0.5	-0.52	-0.43	-1.93	-1.09	1.44	1.89
	32 / 0.5	-0.55	-1.22	-1.00	1.98	2.39	2.89
**FOF / ZDV**	128 / 0.015	**-2.02**	**-5.31**	**-7.26**	-2.59	**-3.44**	**-3.44**
	32 / 0.015	0	-0.53	-0.87	-0.02	2.35	3.11

AMK, amikacin; ATM, aztreonam; AZM, azithromycin, BLE, bleomycin; BUT, tertbutaline; CDR, cefdinir; CRO, ceftriaxone; CST, colistin; DOX, doxycycline; DOR, doripenem; ENR, enrofloxacin; FOF, fosfomycin; FZD, furazolidone; IBN, ibandronate; IVM, ivermectin; LVX, levofloxacin; LMF, lomefloxacin; MET, methacycline; MOX, moxalactam; PFD, penfluridol; PRA, pralidoxime; RAD, cephradine; RFX, rifaximin; STP, streptomycin; TCS, triclosan; TGC, tigecycline; TMP, trimethoprim; TOB, tobramycin; ZDV, zidovudine

.

Table S7. Strain characterization of *K. pneumoniae* strains and susceptibility profile to drugs evaluated in this study. Clinical categorization according to EUCAST breakpoints are displayed in brackets. MIC values were obtained by broth microdilution method in CAMHB. For CAZ-AVI and FOF MIC determination, medium was supplemented with 4 mg/L of avibactam and 25 mg/L of glucose-6-phosphate respectively.

				MIC (mg/L)							
**Isolate**	**Resistance mechanism**	**Source**	**MDR/XDR**	**CST**	**FOF**	**TGC***	**ETP**	**MEM**	**CAZ-AVI**	**ZDV**	**AZM**
E-1	CTX-M 14	Rectal swab	XDR	0.5 (S)	>64 (R)	4 (R)	>32 (R)	8 (I)	1 (S)	0.25-0.5	8
E-2	CTX-M 15	Blood	MDR	0.5 (S)	>64 (R)	0.5 (S)	64 (R)	4-8 (I)	1 (S)	0.5-1	8
E-3	CTX-M 15	Abscess	MDR	1-2 (S)	>64 (R)	4 (R)	16 (R)	2-4 (I)	1 (S)	2	8
E-4	CTX-M 15	Blood	MDR	0.5 (S)	>64 (R)	4 (R)	1 (R)	0.03 (S)	0.5 (S)	1	8
E-5	SHV-1 + porin loss	Blood	MDR	0.5 (S)	8 (S)	0.5-1 (S)	0.25 (S)	0.03 (S)	0.06-0.12 (S)	0.5-1	8-16
A-6	AmpC ACT-1	SEIMC CCS07	MDR	≤0.5 (S)	>64 (R)	1-2 (R)	4-8 (R)	0.5 (S)	0.5 (S)	8-16	8
C-7	OXA-48	Blood	MDR	1 (S)	>64 (R)	2 (R)	8-16 (R)	4 (I)	0.5 (S)	2	4-8
CS-8	Colistin R	Urine	MDR	16 (R)	>64 (R)	1 (R)	0.5 (S)	0.5-1 (S)	0.5 (S)	0.5	8
CSE-9	VIM-1 + CTX-M 15 + colistin R	SEIMC CCS04	XDR	16 (R)	>64(R)	1-2 (R)	8-16 (R)	16-32 (R)	>64 (R)	≥64	64
CE-10	CTX-M 15 + OXA-48	Blood	MDR	1-2 (S)	>64 (R)	1-2 (R)	8 (R)	4 (I)	0.25 (S)	64	4
CEE-11	KPC-3 + SHV-11 + TEM-1	SEIMC CCS05	XDR	2 (S)	>64 (R)	4 (R)	>64 (R)	>64 (R)	4 (S)	0.5-1	≥64
CSEE-12	OXA-1 + SHV-1 + colistin R	EARS QC	MDR	4 (R)	64 (R)	1 (R)	8-16 (R)	1-2 (S)	0.5 (S)	1	8

MDR: non-susceptible to ≥1 agent in ≥3 antimicrobial categories; XDR: non-susceptible to ≥1 agent in all but ≤2 categories (categorization according to susceptibility results provided in Table S2); CAZ-AVI, ceftazidime-avibactam; CST, colistin; FOF, fosfomycin; ETP, ertapenem; MEM, meropenem; TGC, tigecycline; ZDV, zidovudine; AZM, azithromycin.

*EUCAST clinical breakpoints for tigecycline are only applied to *Escherichia coli* and *Citrobacter koseri*

EARS QC, European Antimicrobial Resistance Surveillance Quality Control; R, resistant; S, susceptible; S*: susceptible, increased exposure; SEIMC: Spanish Society of Infectious Diseases and Clinical Microbiology
